# When Mathematical Learning Experiences Match Self-Efficacy Beliefs: Content Specificity and Cognitive Performance Across 49 Education Systems

**DOI:** 10.3390/jintelligence14100263

**Published:** 2026-10-16

**Authors:** Xin Liu

**Affiliations:** College of Teacher Education, Ningbo University, Ningbo 315211, China; liuxin2@nbu.edu.cn

**Keywords:** mathematics self-efficacy, content specificity, informational proximity, mathematics learning, cognitive activation, digital inquiry, cognitive performance, cross-system generalizability

## Abstract

Self-efficacy is task-referenced, yet it is often treated as a single subject-level belief. Drawing on the specificity and correspondence principles of self-efficacy theory, this study asks whether learning experiences that more closely match a judged capability show stronger and more generalizable relations with content-specific mathematics self-efficacy. PISA 2022 data from 173,641 adolescents in 49 education systems were analyzed. Five learning experiences ranged from direct task exposure to process-focused cognitive activation and context-mediated digital inquiry. Item-level analyses supported the intended within-domain distinctions, while exact loading comparability was less consistent for task exposure. System-specific weighted structural models accounted for school clustering and all ten mathematics plausible values, and the resulting estimates were synthesized with random-effects meta-analysis. Direct task exposure produced the clearest pattern. Formal/applied exposure was more strongly associated with formal/applied self-efficacy, whereas reasoning/21st-century exposure was more strongly associated with reasoning-oriented self-efficacy. The combined task-matching summary was positive in all 49 systems, and its random-effects prediction interval remained above zero. Cognitive activation showed differentiated but nonliteral correspondence, while digital inquiry was positively associated with both beliefs and varied more across systems. Both self-efficacy dimensions were positively associated with mathematics performance when examined separately, although their unique criterion relations differed in joint models. The findings show that mathematics self-efficacy has meaningful within-domain structure and that experience–belief correspondence depends on how closely learning experiences instantiate the capability being judged.

## 1. Introduction

Mathematics performance reflects several sources of individual difference. Cognitive ability matters, but so do acquired knowledge, opportunities to learn, and students’ engagement with mathematically demanding activity (Deary et al., 2007; Hischa et al., 2026; Niileksela et al., 2026). Domain-general abilities and mathematics-specific skills can make partly distinct contributions to achievement, and their relative importance changes across development (Fiedler et al., 2025; Hischa et al., 2026). An account of mathematics performance is therefore incomplete if it considers cognitive ability alone.

Self-efficacy adds a different layer of explanation. It is a judgment about one’s capability to organize and carry out the actions required for a particular task or course of action (Bandura, 1997; Pajares, 1996). It is therefore conceptually distinct from intelligence or cognitive ability. Ability concerns what a person can do under specified conditions; self-efficacy concerns what that person believes he or she can do. The distinction does not make self-efficacy peripheral. Capability judgments can shape task choice, persistence, and strategy use when students encounter difficulty (Bandura, 1997; Schunk & DiBenedetto, 2020). Cognitive resources and self-belief can thus make separable but complementary contributions to academic performance (Almeida et al., 2026).

The level at which self-efficacy is defined is critical. Mathematics is a school subject, but it is not one kind of activity. Applying a familiar procedure, interpreting a representation, constructing a model, and reasoning through an unfamiliar problem draw on overlapping but nonidentical knowledge and strategies. Students may therefore feel capable in one kind of mathematical work and less certain in another (Bong, 2001; Bong & Skaalvik, 2003). Earlier research supports this within-domain specificity: exposure to particular mathematics tasks is most strongly associated with efficacy judgments that refer to similar tasks (Borgonovi & Pokropek, 2019). Treating mathematics self-efficacy as a single subject-level belief may therefore hide psychologically meaningful differentiation.

That specificity raises a second question: which learning experiences are most informative for a particular capability judgment? Self-efficacy theory treats experience as information that must be interpreted, not as a simple exposure count (Bandura, 1997; Usher & Pajares, 2008). Some experiences closely reproduce the demands named in an efficacy judgment. Others provide broader information about how students reason, participate, or use tools. The framework developed here uses informational proximity as an organizing proposition for this difference. It does not treat proximity as a directly measured psychological mechanism. Direct encounters with closely matched tasks should require relatively little inferential translation to inform a capability judgment. Broader instructional or technology-mediated experiences may be more dependent on classroom organization, guidance, feedback, and students’ interpretation.

This distinction offers a way to organize learning experiences that are often treated as interchangeable predictors. Task exposure provides a direct content match. Cognitive activation instead concerns mathematical processes such as explaining, comparing, connecting, and reasoning (Baumert et al., 2010; Praetorius et al., 2018/2023). Digital inquiry is more context-mediated because the same technology can support exploration and feedback or simply move routine work onto a screen (Hillmayr et al., 2020; Rakes et al., 2020; Ran et al., 2022). These experiences may therefore differ not only in the size of their associations with self-efficacy but also in how specifically and how consistently they correspond to the capability being judged.

A third issue is whether differentiated efficacy beliefs have distinct external meaning. Closely related self-efficacy dimensions may both correlate positively with mathematics performance because they share a broad mathematics-capability component. Separate models cannot show whether either belief carries unique criterion-related information. Joint models can. Cross-system analysis adds another test of scope: a relation grounded in close task-demand correspondence may recur broadly, whereas a relation whose meaning depends more on instructional organization or technology use may be more context-dependent.

The Programme for International Student Assessment (PISA) 2022 provides the measures needed to examine these questions at scale. It distinguishes formal/applied from reasoning/21st-century mathematics self-efficacy and includes corresponding task-exposure measures, two forms of cognitive activation, and digitally supported inquiry (OECD, 2023, 2024a). The present study analyzes 173,641 adolescents in 49 education systems. It first evaluates whether the paired constructs are empirically distinguishable and sufficiently comparable at the measurement level. It then tests whether experience–belief correspondence follows the proposed proximity logic, whether the two self-efficacy dimensions show distinct relations with mathematics performance, and which associations generalize across systems.

## 2. Theoretical Framework

### 2.1. Self-Efficacy as a Content-Specific Capability Judgment

Self-efficacy is a judgment of capability for a defined action, not a general estimate of intelligence, competence, or self-worth (Bandura, 1997; Pajares, 1996). Its explanatory value depends on the level at which the judgment is framed. Broad beliefs can be useful for broad criteria, but self-efficacy becomes more informative when the task demands named in the judgment are more specific (Pajares, 1996). This task-referenced character is central to the construct. It implies that students can hold different efficacy beliefs within the same school subject when activities require different combinations of knowledge, representation, reasoning, and application.

Mathematics is especially suited to this distinction. Applying a familiar procedure, interpreting a representation, constructing a model, and reasoning through an unfamiliar problem all belong to mathematics, but they do not pose identical demands. A subject-level belief may therefore conceal meaningful within-domain differentiation (Bong, 2001; Bong & Skaalvik, 2003). The first premise of the framework is structural: formal/applied and reasoning-oriented self-efficacy should be related because both concern mathematics, yet they should remain empirically distinguishable if students organize capability judgments around task content. This premise is examined at the item level before the experience–belief relations are interpreted.

### 2.2. Learning Experience as Capability Information

Self-efficacy develops through the interpretation of experience. Mastery experience is often a powerful source because successful action can provide direct evidence of capability. Yet the same experience can be interpreted differently depending on difficulty, feedback, prior expectations, and context (Bandura, 1997; Bjerke et al., 2026; Usher & Pajares, 2008). Exposure is therefore not the same as mastery. The key point for the present study is narrower: learning experiences differ in how directly they speak to the capability named in an efficacy judgment.

Three concepts are distinguished here. Task specificity concerns the level and content at which an efficacy judgment is defined. Mastery experience concerns interpreted evidence of successful performance and is an established source of self-efficacy. Informational proximity concerns how closely the demands instantiated by a learning experience resemble the demands named in a capability judgment. A highly proximal experience need not be a mastery experience because students can encounter a closely matched task without succeeding at it. Conversely, a successful but broadly framed experience may inform efficacy without referring to one narrowly defined capability. Informational proximity therefore organizes learning experiences according to how directly they can inform a given judgment. It is not proposed as a new source or psychological mechanism of self-efficacy.

### 2.3. A Proposed Informational-Proximity Ordering Across Mathematical Learning Experiences

Task exposure provides the most direct case. Repeated encounters with formal/applied tasks give students information about handling formal/applied demands. Encounters with reasoning/21st-century tasks provide analogous information about reasoning-oriented demands. Prior evidence that exposure to particular mathematics tasks is most strongly associated with self-efficacy for similar tasks supports this principle (Borgonovi & Pokropek, 2019). The framework therefore expects matched associations to exceed mismatched associations. This is a correspondence claim, not a mastery claim. Frequency of exposure can be informative without establishing successful performance, and the present study does not measure informational proximity as a separate variable.

Cognitive activation is less directly tied to a specific task class. Practices such as explaining solutions, comparing strategies, connecting ideas, and reasoning about mathematical structure expose students to demanding processes that can support more than one kind of task (Baumert et al., 2010; Kunter et al., 2013; Praetorius et al., 2018/2023). The two forms of cognitive activation may therefore produce different association profiles without mapping literally onto labels such as reasoning or formal/applied. Recent cross-system work using PISA 2022 likewise shows that the two cognitive-activation dimensions can relate differently to mathematics outcomes (Liu et al., 2024b). Their relative pattern is therefore treated as an empirical question.

Digitally supported inquiry is more context-mediated. Technology can support exploration, representation, data collection, repeated trials, and feedback, but those functions do not carry a fixed mathematical meaning (Hillmayr et al., 2020; Rakes et al., 2020; Ran et al., 2022). The same digital activity can support conjecture and revision in one classroom and routine completion in another. Inquiry also depends on guidance (Lazonder & Harmsen, 2016). Digital inquiry may therefore inform both self-efficacy dimensions. A modest relative advantage for reasoning-oriented self-efficacy is plausible because inquiry often involves interpretation and exploration, but any differential association should be less stable than direct task-content matching.

### 2.4. Criterion Correspondence and Cross-System Generalizability

Content specificity should also appear in criterion correspondence. Self-efficacy is consistently associated with academic performance, but perceived capability and demonstrated competence are not interchangeable (Honicke & Broadbent, 2016; Multon et al., 1991). Cross-country evidence also shows that confidence and mathematics competence can be systematically miscalibrated (Yang Hansen et al., 2024). If two mathematics self-efficacy dimensions are substantively distinct, each may correspond differently to mathematics performance. Separate models estimate each belief’s overall adjusted relation with the criterion. A joint model asks whether either belief retains unique criterion-related variance after their shared mathematics-capability component is controlled. Because performance can shape later self-efficacy just as self-efficacy can shape later engagement, these associations are interpreted as correspondence rather than as causal effects (Grigg et al., 2018; Holenstein et al., 2022).

Cross-system replication provides a second test of the framework’s scope. A relation rooted in close task-demand correspondence should depend less on how an education system labels or organizes instruction than a relation whose meaning is mediated by classroom practice, technology use, or frames of reference. Cross-national measurement work on mathematics instructional quality also shows that instructional constructs can vary across systems and respondent perspectives even when a common structure is broadly defensible (Liu et al., 2024a). The present argument does not require the literal invariance of every parameter. It makes a more modest generalizability claim: direct task-content matching should recur more consistently, whereas process-focused and context-mediated relations may show greater heterogeneity (Chiu & Klassen, 2010; Usher & Pajares, 2008). Prediction intervals and system-specific contrasts are therefore substantively informative, not merely descriptive statistics.

### 2.5. The Present Study

The theoretical argument yields its clearest directional expectation for direct task exposure. Formal/applied and reasoning/21st-century task exposures closely mirror the demands represented in the corresponding self-efficacy judgments. Each form of exposure should therefore be more strongly associated with its content-matched self-efficacy dimension than with the alternative dimension, producing a positive overall task-matching pattern. Cognitive activation and digitally supported inquiry provide less direct forms of capability-relevant information. They concern broader ways of thinking, participating, and working in mathematics rather than repeated experience with a particular class of tasks. Their associations with the two self-efficacy dimensions may therefore be differentiated without following a simple one-to-one pattern and may depend more strongly on educational context.

Against this background, three substantive research questions guide this study. Before addressing them, this study examines whether the focal dimensions are empirically distinguishable at the item level and how stable their loading structures are across education systems. These item-level analyses provide complementary evidence about construct organization; they do not reconstruct the official PISA scores or directly establish invariance of the published WLE metrics used in the structural models.

**RQ1.**  *Do students’ experiences with formal/applied and reasoning/21st-century mathematics tasks correspond more closely to confidence in the same type of mathematics, and is this pattern evident across education systems?*

**RQ2.**  *How are cognitively activating mathematics teaching and digitally supported inquiry related to students’ confidence in formal/applied and reasoning/21st-century mathematics, and do these relationships show similar patterns across education systems?*

**RQ3.**  *Are formal/applied and reasoning/21st-century mathematics self-efficacies differently related to students’ mathematics performance, and does each remain relevant when the other is taken into account?*

## 3. Methods

### 3.1. Data Source and Analytic Sample

This study used the public-use database from the PISA 2022. The PISA uses a two-stage sampling design in which schools are sampled first and students are sampled within schools. The target population is 15-year-old students enrolled in educational institutions. Mathematics performance is represented by plausible values from the PISA scaling model. These values are multiple draws from a proficiency distribution for population-level inference rather than individual raw scores (OECD, 2024b). In this study, they serve as a domain-specific mathematics performance criterion.

Education systems were screened in the international database before analysis. A system was retained when both self-efficacy indices, all five learning-experience indices, socioeconomic status, gender, the final student weight, the school identifier, and mathematics plausible values were available. Retention also required more than 500 students with valid data on every focal variable. The final structural-model sample included 173,641 students in 49 education systems. System-specific samples ranged from 1487 students in Malta to 14,819 in Spain, with 45 to 959 sampled schools. Supplementary Table S1 lists the systems, student counts, and school clusters. Within each system, the same analytic sample was used across all four structural-model families.

### 3.2. Measures

The structural analyses used the published PISA 2022 weighted-likelihood-estimate (WLE) indices rather than reconstructing scale scores. Table 1 defines each construct and its role in the analysis. Higher values indicate greater reported exposure, stronger perceived capability, or higher socioeconomic status. The learning-experience and self-efficacy indices are continuous. Mathematics performance was analyzed with all ten plausible values (OECD, 2023, 2024a, 2024b). Item-level multigroup confirmatory factor analyses were conducted separately to evaluate measurement structure and cross-system loading comparability, as described in Section 3.3. The item-level models and the published WLE indices therefore serve related but distinct purposes: the former evaluate construct organization, whereas the latter are the official PISA scores used in the structural analyses.

### 3.3. Item-Level Measurement Models and Measurement Invariance

Although the structural analyses used the published PISA 2022 indices, item-level multigroup confirmatory factor analyses (MGCFAs) were conducted across the same 49 education systems. These analyses served two purposes. First, they tested whether the paired constructs were empirically distinguishable. Second, they examined how stable the item-loading structure was across systems. They were not intended to reproduce the OECD international IRT scaling or to validate the invariance of the published WLE metrics themselves. Four measurement blocks were examined: (a) formal/applied and reasoning/21st-century mathematics self-efficacy (9 and 10 items), (b) exposure to formal/applied and reasoning/21st-century mathematics tasks (9 and 10 items), (c) foster-reasoning and encourage-mathematical-thinking cognitive activation (9 items each), and (d) digital inquiry (10 items). For the first three blocks, the theorized two-factor model was compared with a one-factor alternative.

Models were estimated in Mplus 9.1 with the final student weight and an adjustment for school clustering. Several focal PISA 2022 scales used planned within-construct matrix sampling. To retain the available item information under a common full-information likelihood framework, the four- and five-category items were treated as approximately continuous and estimated with robust maximum likelihood. Simulation evidence indicates that robust continuous estimation can perform reasonably with five response categories and, under some distributional conditions, with four, although categorical estimation can be preferable when categories are sparse or highly nonnormal (Rhemtulla et al., 2012). We therefore treat the MLR specification as a pragmatic approximation rather than as a general claim that ordered items are continuous. The formal/applied and reasoning/21st-century task-exposure items were reverse-coded so that higher values represented more frequent exposure. Because the item-level analyses used all available respondents in the selected systems, sample sizes varied across blocks (N = 308,080–316,018) and were larger than the structural-model sample.

Initial configural models were examined for recurrent local dependence. Modification indices and STDYX expected parameter changes (EPCs) were inspected across systems. The screening values were pragmatic safeguards rather than universal cutoffs. An MI of at least 10 served only as an initial flag; a residual covariance was retained only when the same item pair met that threshold in at least 40 of the 49 systems, the median absolute STDYX EPC was at least 0.20 with a consistent direction, and the shared item content or response format provided a substantive explanation. The recurrence requirement was deliberately stringent so that local dependence observed in only one or a few systems would not alter the common specification. The resulting factor pattern and residual-covariance structure were then held constant across systems, while residual-covariance values remained free. The corresponding one- and two-factor comparison models retained the same residual-covariance structure, so differences in dimensional fit were not produced by giving one-factor-solution additional local-dependence parameters. This procedure was used to represent replicated local dependence rather than to improve fit mechanically (Yoon & Millsap, 2007).

Model fit was evaluated jointly with the comparative fit index (CFI), Tucker–Lewis index (TLI), root-mean-square error of approximation (RMSEA), and standardized root-mean-square residual (SRMR). Values around CFI/TLI ≥ 0.95, RMSEA ≤ 0.06, and SRMR ≤ 0.08 were treated as guides for close fit (Hu & Bentler, 1999), not as rigid pass/fail rules. Measurement invariance was then examined by constraining factor loadings across systems (metric model) and subsequently constraining item intercepts as well (scalar model). As conventional guides, deterioration no greater than 0.010 in the CFI, 0.015 in the RMSEA, and 0.030 in the SRMR was considered supportive of metric invariance; for scalar invariance, the SRMR guide was 0.010 (Chen, 2007). These criteria were interpreted cautiously because they were developed in settings with far fewer groups, whereas large-scale international MGCFAs can behave differently when many unequally sized groups are analyzed (Rutkowski & Svetina, 2014). Fit changes were therefore interpreted jointly with absolute fit, parameter admissibility, and the purpose of the comparison. Because MLR was used, the one- and two-factor models were compared descriptively rather than with an uncorrected chi-square difference test. Full scalar invariance was tested as a robustness check, but partial scalar models were not pursued because latent mean comparisons were outside the scope of this study. Detailed fit and invariance results are reported in Supplementary Table S6; retained residual covariances are reported in Table S7.

Two additional measurement sensitivities were conducted for the revision. First, the intended factor structures were re-estimated with the WLSMV while treating the four- and five-category indicators as ordered-categorical. These models deliberately omitted the locally correlated residuals retained in the primary MLR specification, providing a conservative check of whether the intended dimensional structures remained recoverable under an ordinal estimator. This sensitivity was not used to make new metric- or scalar-invariance claims. Second, the primary MLR measurement models were re-estimated after removing all retained residual covariances. This second check isolated the extent to which dimensional fit and loading-invariance conclusions depended on the local-dependence terms. Results are reported in Supplementary Tables S10 and S11.

### 3.4. System-Specific Model Specification

Models were estimated separately for each education system in Mplus 9.1 (Muthén & Muthén, 2017). The final student weight (W_FSTUWT) was applied, and standard errors were adjusted for school clustering through CNTSCHID. Estimation used robust maximum likelihood with TYPE = COMPLEX. All ten mathematics plausible values were analyzed with TYPE = IMPUTATION, which fits the model to each plausible-value file and combines estimates using multiple-imputation rules. To retain full-information likelihood treatment for the learning-experience indices, these variables were modeled as endogenous and regressed on socioeconomic status and gender.

The primary model contained three parallel outcomes: formal/applied self-efficacy (MATHEFF), reasoning/21st-century self-efficacy (MATHEF21), and mathematics performance (PVMATH). Each outcome was regressed on digital inquiry, the two cognitive-activation indices, the two task-exposure indices, socioeconomic status, and gender. Residual covariances were freely estimated among the five learning experiences and among the three outcomes. Self-efficacy was not specified as a mediator because all focal variables were measured in the same assessment cycle. The model was saturated (df = 0), so global fit indices were not interpreted.

Five paired-path contrasts tested whether the same learning experience related differently to the two self-efficacy dimensions. D_ICT_, D_RCO_, and D_MCO_ were defined as the MATHEF21 coefficient minus the MATHEFF coefficient. D_EFA_ was coded in the opposite direction (MATHEFF minus MATHEF21), so a positive value represented a formal/applied match. D_E21_ was defined as MATHEF21 minus MATHEFF. TASKMAT was the mean of D_EFA_ and D_E21_ and served as the overall task-matching summary on the published WLE metrics. Within each system, a five-degree-of-freedom Wald test examined whether the five paired differences were jointly zero.

Formal contrasts used unstandardized paths because their standard errors depend on the estimated covariance between paired coefficients. STDYX coefficients were extracted separately to describe association magnitude. Because TASKMAT averages contrasts involving two different published task-exposure indices, a scale-free descriptive sensitivity was also calculated from the system-specific STDYX pathways:

$$\begin{array}{l} D_{EFA,std}\;=\;\beta (\mathrm{EXPOFA}\;\to \;\mathrm{MATHEFF})\;-\;\beta (\mathrm{EXPOFA}\;\to \;\mathrm{MATHEF}21),\\ D_{E21,std}\;=\;\beta (\mathrm{EXPO}21\mathrm{ST}\;\to \;\mathrm{MATHEF}21)\;-\;\beta (\mathrm{EXPO}21\mathrm{ST}\;\to \;\mathrm{MATHEFF}),\;and\\ {\mathrm{TASKMAT}}_{\mathrm{std}}\;=\;(D_{EFA,\mathrm{std}}\;+\;D_{E21,\mathrm{std}})/2. \end{array}$$

These standardized differences were used only to check the direction and scale robustness of the combined task-matching pattern; formal *p* values were not attached because the covariance between paired STDYX coefficients was not available from the extracted output. Explained variance was summarized with R^2^, and incremental explanatory contribution in the performance models was expressed as ΔR^2^ relative to the baseline model. No additional Cohen-style small/medium/large thresholds or f^2^ transformations were imposed because standardized path magnitudes and changes in explained variance are model-dependent when predictors are correlated.

For a diagnostic assessment of the shared variance among the five learning-experience predictors, the system-specific residual correlations from the primary models were also extracted. These correlations are adjusted for the ESCS and gender because the five learning-experience variables were modeled as endogenous outcomes of those covariates. Correlations were synthesized across systems on the Fisher z scale with random-effects models and then back-transformed to r. This diagnostic addresses predictor overlap; it is not used as a separate substantive outcome. Results are reported in Supplementary Table S9.

### 3.5. Supplementary Performance Models

Three additional model families examined self-efficacy–performance correspondence. Model 2 added MATHEFF to the mathematics performance equation while retaining the five learning experiences and covariates. Model 3 replaced MATHEFF with MATHEF21. Model 4 entered both self-efficacy dimensions together and tested whether their unstandardized coefficients were equal. The separate models describe each belief’s adjusted relation with performance when entered alone. The joint model describes the unique relation of each belief after the shared variance is controlled. Overall, PVMATH was retained as the broad external domain criterion specified in RQ3. More fine-grained PISA process indicators organize performance around formulating, employing, interpreting/evaluating, and reasoning, whereas MATHEFF and MATHEF21 distinguish broad classes of mathematical task content; these classifications do not map one to one. Such process indicators were therefore not treated as direct matched validation criteria in the present analysis. These comparisons are interpreted as evidence about overlap and broad criterion correspondence, not as process-specific matching, mediation, or causal influence.

### 3.6. Cross-System Random-Effects Synthesis

The education system was the unit of synthesis. For each formal contrast and standardized pathway, system-specific estimates and standard errors were combined with inverse-variance random-effects models. Between-system variance was estimated with the Paule–Mandel method (Paule & Mandel, 1982), while confidence intervals used the Hartung–Knapp adjustment (IntHout et al., 2014). The synthesis reports pooled estimates, 95% confidence intervals, 95% prediction intervals, tau-squared, I-squared, Cochran’s Q, and the number of systems with significant positive, negative, or nonsignificant estimates. Prediction intervals are emphasized because they show the likely range of the underlying association in another education system rather than only the precision of the pooled average.

Data management and synthesis were conducted in Python 3.13 with pandas, NumPy, and SciPy. Mplus 9.1 was used for model estimation. Random-effects calculations were checked against the conventional formulations described by Viechtbauer (2010). For TASKMAT, the synthesis was repeated after omitting each system in turn. Exploratory system-level meta-regressions were not fitted, and no moderators were specified in advance. With 49 systems, broad post hoc cultural or policy explanations would not be well supported.

### 3.7. Complex Sampling and Inferential Scope

The analyses incorporated the final student weight, school clustering, robust estimation, and all ten plausible values. The 80 PISA replicate weights were not present in the analytic files used for the structural models, so sampling variances were estimated with the school-clustered robust estimator rather than the full OECD balanced-repeated-replication procedure (OECD, 2024b; Rutkowski et al., 2010). These approaches are not equivalent. The final weight preserves the weighted point estimation used here, and clustering accounts for within-school dependence, but the absence of replicate-weight BRR creates additional uncertainty around system-specific standard errors, *p* values, and the exact number of systems classified as statistically significant. Accordingly, the revised interpretation gives primary weight to coefficient direction and magnitude, random-effects pooled estimates, heterogeneity, and prediction intervals; counts of significant systems are treated as secondary descriptive evidence under the primary variance estimator. All focal variables were measured at one time point, so the models estimate conditional associations and cannot establish development, causation, or mediation.

## 4. Results

### 4.1. Measurement Structure and Cross-System Comparability

The item-level analyses supported the intended within-domain distinctions (Supplementary Table S6). For self-efficacy, the two-factor model (CFI = 0.961, TLI = 0.955, RMSEA = 0.027, SRMR = 0.046) fit substantially better than the one-factor alternative (CFI = 0.889, TLI = 0.871, RMSEA = 0.046, SRMR = 0.071). The same pattern appeared for task exposure (two-factor: CFI = 0.936, TLI = 0.925, RMSEA = 0.031, SRMR = 0.059; one-factor: CFI = 0.847, TLI = 0.820, RMSEA = 0.049, SRMR = 0.081) and cognitive activation (two-factor: CFI = 0.945, TLI = 0.933, RMSEA = 0.036, SRMR = 0.060; one-factor: CFI = 0.818, TLI = 0.781, RMSEA = 0.064, SRMR = 0.103). The one-factor digital-inquiry model also showed good overall fit (CFI = 0.967, TLI = 0.951, RMSEA = 0.054, SRMR = 0.030).

Across the 49 systems, latent correlations between paired factors were substantial but consistently below unity. Self-efficacy correlations ranged from 0.635 to 0.886 (median = 0.758), task-exposure correlations from 0.590 to 0.722 (median = 0.658), and cognitive-activation correlations from 0.456 to 0.727 (median = 0.615). The paired dimensions therefore shared meaningful variance but remained empirically distinguishable. Metric invariance was supported by the prespecified fit-change guides for self-efficacy (ΔCFI = −0.005, ΔRMSEA = 0.001, ΔSRMR = 0.010), cognitive activation (ΔCFI = −0.008, ΔRMSEA = 0.000, ΔSRMR = 0.007), and digital inquiry (ΔCFI = −0.008, ΔRMSEA = −0.001, ΔSRMR = 0.018). Task exposure did not meet the prespecified ΔCFI criterion for full metric invariance (ΔCFI = −0.013), although ΔRMSEA (0.002), ΔSRMR (0.017), and absolute metric-model fit remained comparatively stable. We therefore interpret the task-exposure evidence as supporting a stable two-factor organization with some cross-system loading noninvariance, not as establishing full metric invariance. Full scalar invariance was not supported for any block. Because this study does not compare latent means across systems, no partial scalar model was pursued.

The revision sensitivities qualified but did not overturn this measurement interpretation. Under the conservative ordered-categorical WLSMV specification without local residual covariances, the intended factor structures remained estimable for all four blocks: self-efficacy (CFI = 0.971, TLI = 0.967, RMSEA = 0.061, SRMR = 0.084), task exposure (CFI = 0.934, TLI = 0.925, RMSEA = 0.068, SRMR = 0.142), cognitive activation (CFI = 0.982, TLI = 0.979, RMSEA = 0.051, SRMR = 0.057), and digital inquiry (CFI = 0.937, TLI = 0.919, RMSEA = 0.131, SRMR = 0.042). Absolute fit was therefore mixed for task exposure and digital inquiry, but the intended structures were not dependent on treating the indicators as continuous. Separately, removing the retained residual covariances from the original MLR models reduced absolute fit, especially for task exposure and digital inquiry. The metric ΔCFI values in the no-residual models were −0.008 for self-efficacy, −0.021 for task exposure, −0.009 for cognitive activation, and −0.015 for digital inquiry. For the three paired blocks, the no-residual two-factor models also fit descriptively better than the corresponding one-factor alternatives, although the auxiliary one-factor models produced possible-local-solution warnings and are therefore interpreted only descriptively. Together, these checks show that recurrent local dependence materially improves measurement fit, while the basic within-domain distinctions do not arise solely from the retained residual-covariance terms (Supplementary Tables S10 and S11).

All 196 system-specific structural models (four model families across 49 systems) completed estimation for all ten plausible values. No output showed an estimation error, convergence failure, non-positive-definite covariance matrix, or inadmissible residual variance. The same parameterization was used in every system. Under the primary complex-sample variance estimator, the joint five-contrast Wald test was significant in 48 systems. The Dominican Republic was the only exception (χ^2^(5) = 8.53, *p* = .129). Differentiated pathway structure was therefore evident in nearly every system, although the individual contrasts were not equally consistent and the exact system-level significance classifications should be interpreted in light of the replicate-weight limitation described in Section 3.7.

### 4.2. Experience–Belief Correspondence and Content Matching

**Figure 1:**
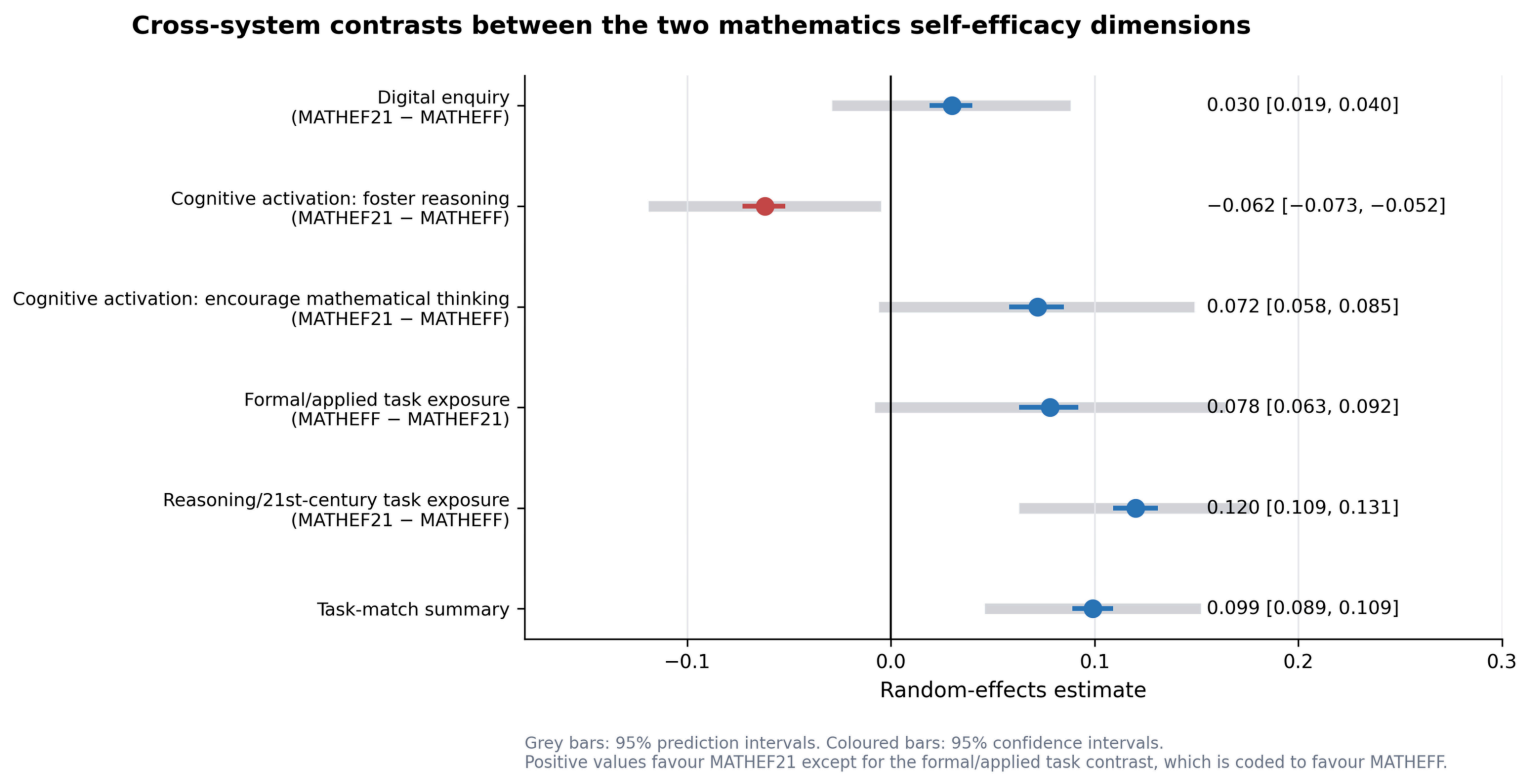
The random-effects synthesis of the five paired-path contrasts and the overall task-matching summary across 49 education systems. Note: Blue points and confidence-interval bars indicate positive pooled contrasts, whereas the red point and confidence-interval bar indicate the negative pooled contrast. Points indicate pooled differences, dark horizontal lines indicate 95% confidence intervals, and light-gray lines indicate 95% prediction intervals; the vertical line denotes zero. MATHEFF—self-efficacy for formal/applied mathematics tasks; MATHEF21—self-efficacy for reasoning/21st-century mathematics tasks. The digital-inquiry and cognitive-activation contrasts are coded as MATHEF21 minus MATHEFF. The formal/applied task-exposure contrast is coded as MATHEFF minus MATHEF21, and the reasoning/21st-century task-exposure contrast as MATHEF21 minus MATHEFF. The overall task-matching summary is the mean of the two task-exposure contrasts after both are oriented as matched minus unmatched.

Table 2 and Figure 1 report the five paired-path contrasts and the overall task-matching summary. The clearest result concerned reasoning/21st-century task exposure. D_E21_ was positive in every system and statistically significant in 48 under the primary variance estimator. The pooled difference was 0.120 (95% CI [0.109, 0.131]), and its 95% prediction interval [0.063, 0.176] remained above zero. The matched advantage therefore extended beyond the pooled average and did not depend on the exact system-level significance count.

Formal/applied task exposure also favored the matching belief. D_EFA_ pooled to 0.078 (95% CI [0.063, 0.092]) and was significantly positive in 32 systems under the primary variance estimator. Its prediction interval [−0.008, 0.164] crossed zero, so the average pattern was clear but less consistent across systems. The two task contrasts were then combined. The overall task-matching summary was 0.099 (95% CI [0.089, 0.109]; prediction interval [0.046, 0.152]) and was positive in all 49 systems. Forty-four systems reached *p* < .05 under the primary cluster-robust variance estimator, but this count is treated as secondary because the analytic files did not contain the 80 PISA replicate weights. Leave-one-system-out pooled estimates ranged only from 0.097 to 0.101.

The scale-free sensitivity produced the same combined directional pattern (Supplementary Table S8). Across the 49 systems, the standardized task-matching summary was positive in every system (mean = 0.092, median = 0.089, range = 0.022–0.150). The standardized reasoning/21st-century matched contrast was positive in all 49 systems, and the standardized formal/applied matched contrast was positive in 46. Because these standardized differences were computed from STDYX point estimates without the covariance needed for a formal difference standard error, they are treated as descriptive sensitivity evidence rather than as replacement tests for the unstandardized contrasts.

The cognitive-activation contrasts showed different relative profiles. The index labeled foster reasoning was more strongly associated with MATHEFF (D_RCO_ = −0.062, 95% CI [−0.073, −0.052]). In contrast, encourage mathematical thinking was more strongly associated with MATHEF21 (D_MCO_ = 0.072, 95% CI [0.058, 0.085]). Digital inquiry showed a smaller average difference favoring MATHEF21 (D_ICT_ = 0.030, 95% CI [0.019, 0.040]), but its prediction interval crossed zero. The digital-inquiry difference was significantly positive in 18 systems and significantly negative in one.

**Figure 2:**
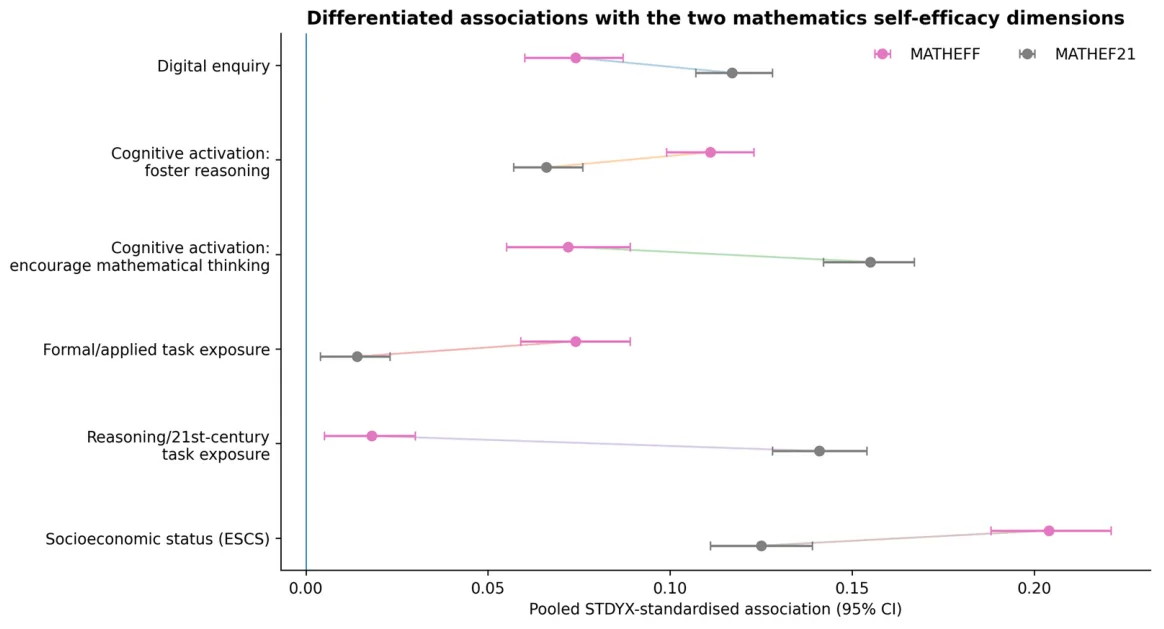
Pooled standardized associations of learning experiences and socioeconomic status with formal/applied mathematics self-efficacy (MATHEFF) and reasoning/21st-century mathematics self-efficacy (MATHEF21). Error bars show 95% confidence intervals. Note: Pink points denote MATHEFF and gray points denote MATHEF21; the connecting lines pair the two estimates for each predictor and are included only as visual guides.

The pooled standardized coefficients clarify the magnitude of these relations (Table 3; Figure 2). Digital inquiry was positively associated with both self-efficacy dimensions, but the association was stronger for MATHEF21 (β = 0.117) than for MATHEFF (β = 0.074). Cognitive activation that encouraged mathematical thinking showed the same relative pattern (β = 0.155 vs. 0.072). In contrast, the foster-reasoning index was more strongly associated with MATHEFF (β = 0.111) than with MATHEF21 (β = 0.066).

Task exposure produced the clearest separation. Formal/applied exposure was associated with MATHEFF at β = 0.074 and with MATHEF21 at β = 0.014. Reasoning/21st-century exposure was associated with MATHEF21 at β = 0.141 and with MATHEFF at β = 0.018. Socioeconomic status was positively related to both beliefs, but the pooled coefficient was larger for MATHEFF (β = 0.204) than for MATHEF21 (β = 0.125).

The primary model also estimated mutually adjusted associations between the five learning experiences and mathematics performance. These coefficients were secondary to the primary theory-guided content-matching tests. Several changed sign after correlated predictors were entered together. Diagnostic synthesis of the predictor residual correlations showed the greatest overlap within the two paired domains: r = 0.542 between the two cognitive-activation indices and r = 0.517 between the two task-exposure indices. The remaining pooled residual correlations ranged from 0.091 to 0.275 (Supplementary Table S9). The predictors therefore shared meaningful variance but were not near-redundant. Their mutually adjusted coefficients represent the component of each learning experience remaining after shared variance with the other experiences has been removed and may show suppression-like sign changes. The complete performance pathways are reported in Supplementary Table S3. The clearest positive conditional association was for cognitive activation that fostered reasoning (pooled β = 0.106, 95% CI [0.092, 0.121]). Negative residual coefficients for other experiences are not interpreted as harmful effects.

### 4.3. Self-Efficacy and Mathematics Performance

**Figure 3:**
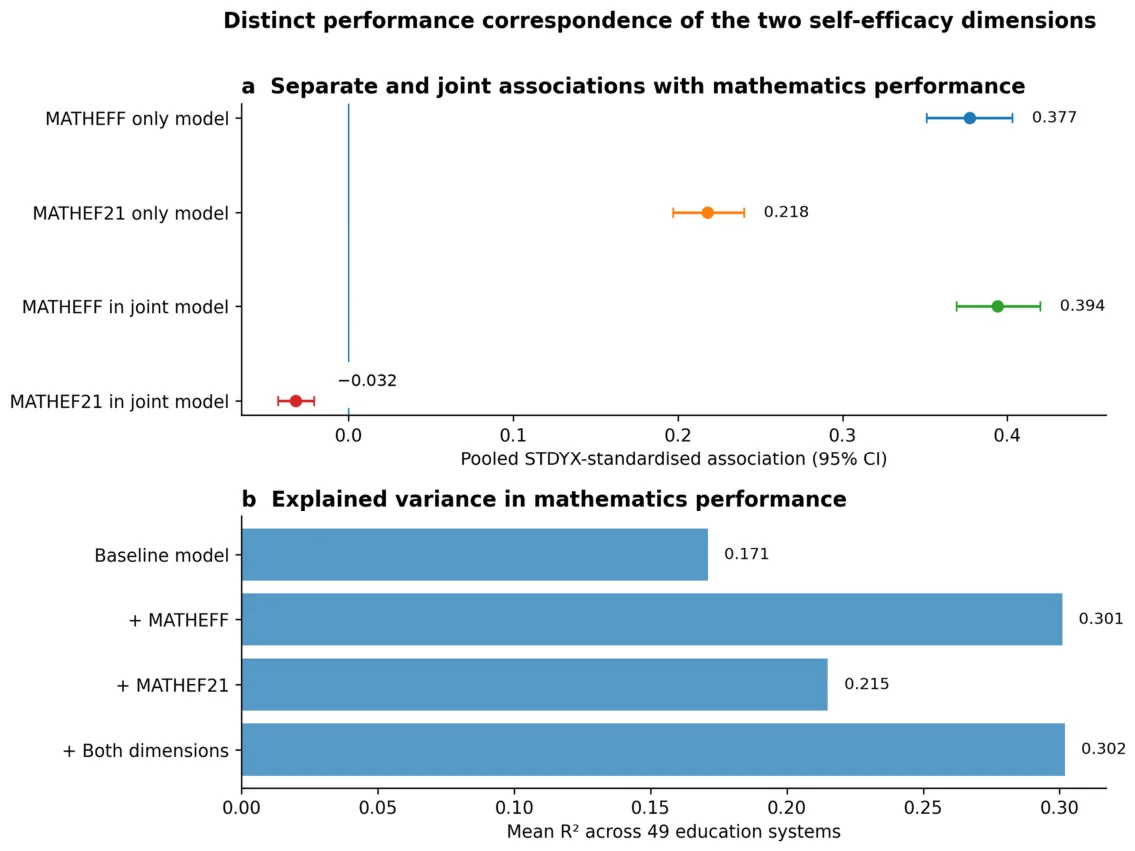
Separate and joint associations of mathematics self-efficacy with mathematics performance and corresponding explained variance across 49 education systems. In panel (**a**), blue, orange, green, and red points denote the MATHEFF-only, MATHEF21-only, MATHEFF joint, and MATHEF21 joint estimates, respectively. Panel (**a**) shows random-effects pooled STDYX coefficients for MATHEFF and MATHEF21 when entered separately and jointly; error bars represent 95% confidence intervals. MATHEFF—self-efficacy for formal/applied mathematics tasks; MATHEF21—self-efficacy for reasoning/21st-century mathematics tasks. Coefficients from the joint model are conditional on the other self-efficacy dimension. In panel (**b**), the plus sign (+) indicates that the named self-efficacy predictor or predictors are added to the baseline model. Panel (**b**) shows the mean PVMATH R^2^ across the 49 system-specific models. The baseline model included the five learning experiences, the ESCS, and gender; the remaining models additionally included MATHEFF, MATHEF21, or both dimensions. Incremental explanatory contribution is reported in Table 4 as ΔR^2^ relative to the baseline model.

When examined separately, both self-efficacy dimensions were positively associated with mathematics performance (Table 4; Figure 3). MATHEFF had a pooled standardized coefficient of 0.377 (95% CI [0.351, 0.403]; prediction interval [0.201, 0.553]) and was significant in all 49 systems. MATHEF21 had a smaller coefficient of 0.218 (95% CI [0.197, 0.240]; prediction interval [0.072, 0.365]) and was significantly positive in 48 systems.

The joint model separated shared from unique associations. MATHEFF retained a strong positive coefficient (β = 0.394, 95% CI [0.369, 0.420]). MATHEF21, by contrast, changed from a positive coefficient when entered alone (β = 0.218) to a small negative coefficient in the joint model (β = −0.032, 95% CI [−0.043, −0.021]); its prediction interval crossed zero [−0.087, 0.023]. The two beliefs remained strongly related after the learning experiences and covariates were controlled (pooled residual r = 0.613). The joint MATHEF21 coefficient therefore answers a narrower question than the separate model: it represents only the part of reasoning/21st-century self-efficacy that remains after the variance it shares with formal/applied self-efficacy has been partialled out. Thirteen systems showed significant negative estimates, one showed a significant positive estimate, and the remaining estimates were mostly nonsignificant under the primary variance estimator.

Explained variance supports this shared-variance interpretation. The mean mathematics performance R^2^ was 0.171 in the baseline model. It increased to 0.301 after MATHEFF was added (ΔR^2^ = 0.130) and to 0.215 after MATHEF21 was added (ΔR^2^ = 0.044). When both dimensions were entered, the mean R^2^ was 0.302 (ΔR^2^ = 0.131), only 0.001 higher than the MATHEFF-only model. MATHEF21 was therefore informative when considered alone but contributed very little additional average explained variance once MATHEFF was included. The negative joint coefficient is accordingly interpreted as a conditional residual coefficient arising in the context of substantial shared variance, not as evidence that reasoning-oriented self-efficacy is detrimental.

### 4.4. Cross-System Generalizability and Robustness

**Figure 4:**
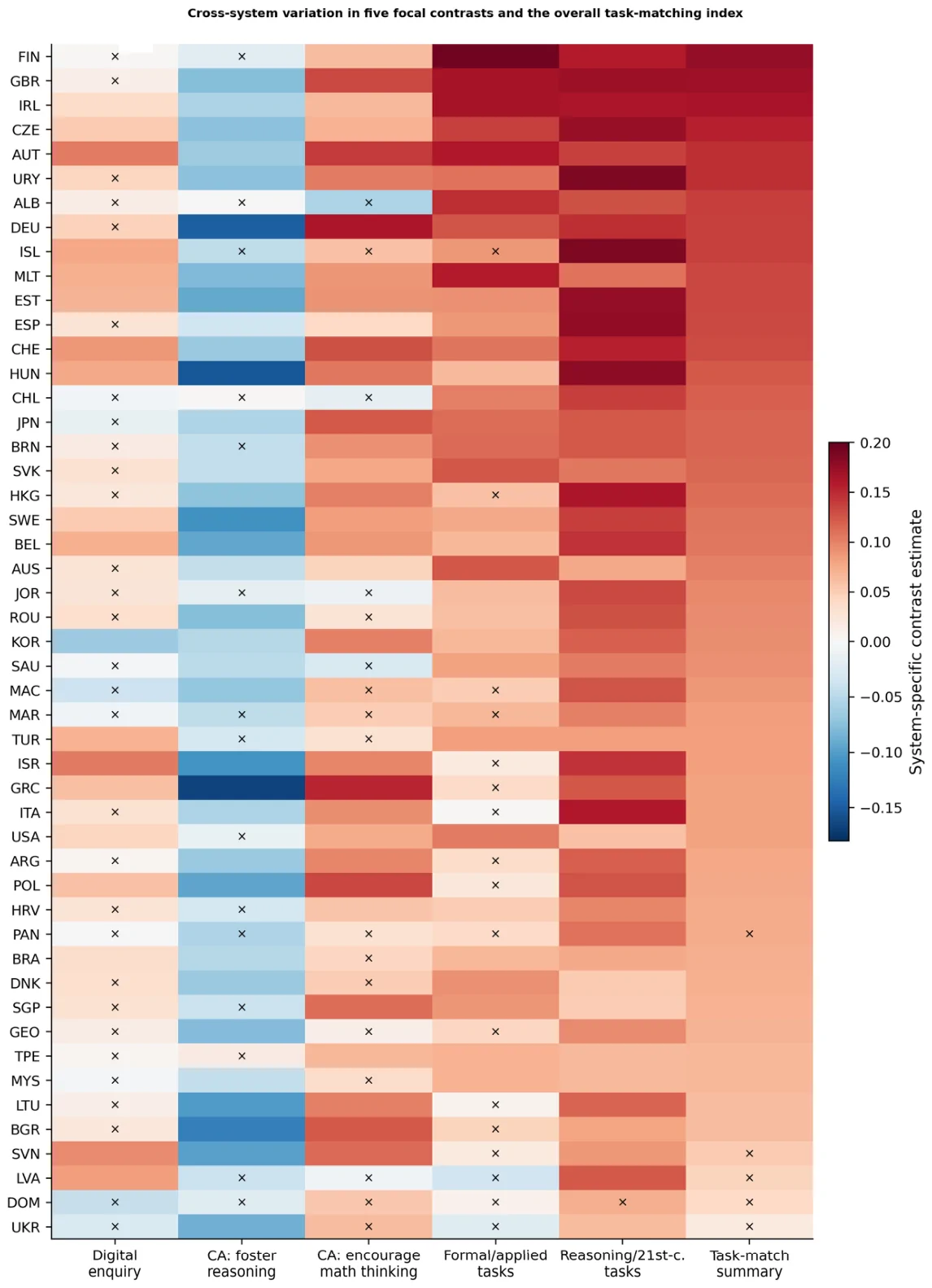
The cross-system variation in the five focal path contrasts and the overall task-matching summary. Columns 1–3 are coded as MATHEF21 minus MATHEFF. The formal/applied task-exposure contrast is coded as MATHEFF minus MATHEF21, whereas the reasoning/21st-century task-exposure contrast is coded as MATHEF21 minus MATHEFF. The final column is the mean of the two task-exposure contrasts after both are oriented as matched minus unmatched. MATHEFF—self-efficacy for formal/applied mathematics tasks; MATHEF21—self-efficacy for reasoning/21st-century mathematics tasks. Warmer colors indicate more positive system-specific contrast estimates and cooler colors more negative estimates; because the coding differs across column blocks, the sign should be interpreted according to the contrast definition for each column. The symbol × indicates that the corresponding within-system contrast was not statistically significant (*p* ≥ .05). Education systems are identified by their PISA three-letter codes and ordered by the overall task-matching summary. The figure displays cross-system generalizability and heterogeneity; it is not intended to rank education systems.

Figure 4 shows the system-specific contrasts alongside the pooled pattern. The overall task-matching summary was positive in every education system. By comparison, the digital-inquiry and cognitive-activation contrasts varied more in magnitude and sometimes in direction. The figure therefore displays generalizability and heterogeneity rather than ranking education systems. No system-level moderators were tested, so the observed variation should not be attributed to particular curricular, cultural, or policy factors.

Four checks reinforce the central task-matching result. First, the task-matching index remained positive in every leave-one-system-out analysis. Second, the matched exposure pattern appeared in both the formal unstandardized contrasts and the pooled standardized pathways. Third, the scale-free descriptive sensitivity based on within-system STDYX pathways produced a positive standardized task-matching summary in all 49 systems (mean = 0.092, median = 0.089, range = 0.022–0.150; Supplementary Table S8). The standardized reasoning/21st-century matched contrast was positive in all 49 systems, and the standardized formal/applied matched contrast was positive in 46. Fourth, the performance models led to the same substantive interpretation: both self-efficacy dimensions were positively related to performance when entered alone, but MATHEFF represented most of their shared performance-related variance when both were entered together. Other findings were less stable. The digital-inquiry contrast had a positive pooled estimate but a prediction interval that crossed zero, and the formal/applied task contrast was not significant in every system. Because replicate weights were unavailable, the exact counts of significant systems should be viewed as secondary to these direction, magnitude, and prediction-interval patterns.

## 5. Discussion

### 5.1. Differentiated Capability Beliefs as the Basis of Content Matching

The measurement analyses support the first premise of the framework while also setting a clear boundary on the cross-system claim. Formal/applied and reasoning/21st-century self-efficacy were strongly related across systems, but the two-factor model fit substantially better than a one-factor alternative. The same pattern held for the paired task-exposure and cognitive-activation constructs. The ordered-categorical sensitivity recovered the intended factor structures, while the no-residual MLR sensitivity showed that the local-dependence terms materially improved absolute fit. Loading invariance was supported for self-efficacy, cognitive activation, and digital inquiry in the primary specification. Task exposure, however, did not meet the prespecified ΔCFI criterion for full metric invariance, and its loading comparability weakened further when recurrent residual covariances were removed. The item-level evidence therefore supports construct differentiation more strongly than the exact equality of all loadings across systems. It should also not be read as establishing invariance of the official PISA WLE scores used in the structural models.

The finding sharpens the meaning of content specificity in self-efficacy theory. Specificity is often presented as a measurement principle: efficacy judgments should correspond to the task or criterion of interest (Bandura, 1997; Pajares, 1996). The results suggest a stronger within-domain implication. Students appear to organize mathematical capability judgments around differences in task demand even when those judgments share a substantial common mathematics component. The structure is neither wholly general nor completely fragmented. It is better described as a correlated system of content-specific beliefs.

This distinction should not be read as evidence that exposure automatically produces self-efficacy. Exposure can create familiarity without creating mastery. Successful experience becomes informative through students’ interpretation of difficulty, progress, feedback, and prior performance (Bandura, 1997; Usher & Pajares, 2008; Zakariya, 2022). The cross-sectional design also leaves temporal ordering unresolved. The measurement evidence therefore does something narrower but important: it shows that the capability beliefs compared in the structural models have a meaningful within-domain organization. The structural estimates themselves are system-specific associations based on the published PISA indices rather than latent path coefficients constrained to equality across systems.

### 5.2. Informational Proximity and the Robustness of Direct Task Matching

The clearest structural finding was the direct match between task experience and capability judgment. Formal/applied exposure was more strongly associated with formal/applied self-efficacy, while reasoning/21st-century exposure was more strongly associated with reasoning-oriented self-efficacy. The overall task-matching summary was positive in all 49 systems, remained positive in every leave-one-system-out analysis, and had a prediction interval above zero. Forty-four systems reached *p* < .05 under the primary cluster-robust variance estimator, but that count is treated as secondary because replicate-weight BRR was unavailable. The scale-free STDYX sensitivity likewise yielded a positive combined task-matching summary in every system. These findings extend earlier evidence on task-exposure specificity (Borgonovi & Pokropek, 2019) and are consistent with the proposed informational-proximity ordering: experiences that most closely instantiate the demands named in a capability judgment show the clearest matched correspondence.

The two direct contrasts were not equally stable. Reasoning/21st-century exposure produced the more uniform matched advantage: its formal contrast was positive in every system, and the standardized sensitivity showed the same direction in all 49 systems. The formal/applied contrast was more heterogeneous, its prediction interval crossed zero, and its standardized counterpart was positive in 46 systems. One possible explanation is that formal and applied work forms part of the routine core of school mathematics in many systems and may therefore provide broader, less diagnostic information about capability. Reasoning/21st-century tasks may be more distinctive because they more often require modeling, interpretation, data use, justification, or unfamiliar problem solving. This explanation remains tentative because curriculum emphasis, task difficulty, and instructional implementation were not tested as system-level moderators. The stronger conclusion is that the combined content-matching pattern generalized broadly, while its two components differed in cross-system stability.

### 5.3. Beyond Direct Matching: Process-Focused and Context-Mediated Experience

The cognitive-activation results show why content specificity should not be reduced to literal label matching. Cognitive activation that fostered reasoning was more strongly associated with formal/applied self-efficacy, not with the reasoning/21st-century belief. Cognitive activation that encouraged mathematical thinking showed the opposite relative pattern. This result is not contradictory within the framework. Process-focused experiences provide information about how students explain, connect, justify, and organize mathematical thinking, and those processes can support several kinds of task. Their meaning for self-efficacy therefore depends on what students actually do and how they interpret the demands, not on the label attached to the instructional practice.

The differentiated cognitive-activation profiles also qualify broad claims that cognitively demanding instruction should have one uniform psychological consequence. Explaining a solution, defending an answer, connecting topics, and applying mathematical logic can make different aspects of capability salient (Baumert et al., 2010; Kunter et al., 2013; Li et al., 2021; Liu et al., 2024b). Content specificity does not mean that every educational experience has one target belief. It means that the informational value of an experience depends on the relation between what the learner is asked to do and what capability is being judged.

Digital inquiry occupied the most context-mediated position in the framework, and its results were correspondingly broad and variable. It was positively associated with both self-efficacy dimensions, with a modest average advantage for reasoning-oriented self-efficacy, but the prediction interval for the relative contrast crossed zero. This pattern fits evidence that digital tools have no fixed educational function. Their value depends on the mathematical activity, guidance, representation, and feedback they support (Hillmayr et al., 2020; Rakes et al., 2020; Ran et al., 2022). The relevant capability information lies not in technology use itself but in the mathematical work that the digital environment makes possible and in how students interpret that work.

### 5.4. Criterion Correspondence, Generalizability, and Educational Meaning

Both self-efficacy dimensions were positively associated with mathematics performance when examined separately, but their unique criterion relations differed in the joint model. Formal/applied self-efficacy retained a strong positive coefficient and accounted for most of the additional explained variance associated with the two beliefs, while reasoning-oriented self-efficacy added little beyond their shared component. Its negative joint coefficient should therefore be interpreted in the context of the pooled residual correlation between the two beliefs (r = 0.613) and the near-identical mean R^2^ for the MATHEFF-only and joint models (0.301 vs. 0.302). The joint coefficient represents a narrow residual component after a closely related efficacy belief is controlled; it does not reverse the clearly positive separate association between MATHEF21 and performance. The result is better understood as shared-variance partitioning than as evidence that reasoning-oriented self-efficacy is detrimental.

This distinction also matters for research on intelligence and cognitive performance. Mathematics performance reflects domain-general cognitive abilities, mathematics-specific skills, prior learning, and opportunities to learn (Fiedler et al., 2025; Hischa et al., 2026; Niileksela et al., 2026). The present study does not treat self-efficacy as intelligence or PISA mathematics performance as a measure of general intelligence. Instead, it identifies a separate layer of task-referenced self-appraisal whose internal differentiation is related both to learning experience and to a domain-specific cognitive criterion. Across systems, the overall task-matching relation was more reproducible than several process-focused and context-mediated contrasts. That pattern is consistent with the informational-proximity account, although it does not by itself prove a universal proximity gradient.

The educational implication is more specific than simply trying to increase confidence. Instruction should make the target capability visible by giving students opportunities to perform the relevant kind of mathematical work and by providing feedback that helps them interpret what successful performance means. Formal/applied capability requires experience with representing, calculating, applying, and checking solutions. Reasoning-oriented capability requires opportunities to justify, model, interpret, compare strategies, and work through unfamiliar situations. Cognitive activation and digital tools are most useful for this purpose when they make the relevant mathematical processes observable, revisable, and open to feedback. Such practices may support more accurate and differentiated capability beliefs, but self-efficacy should complement rather than replace direct assessment of competence.

### 5.5. Limitations and Future Directions

Several limitations constrain the conclusions. First, the data are cross-sectional. Learning experiences may contribute to self-efficacy, but confident students may also select, remember, or interpret experiences differently, and prior performance may influence both. Longitudinal and experimental designs are needed to establish temporal ordering and reciprocal effects. The present study therefore tests correspondence among experience, belief, and performance rather than a causal sequence from experience to self-efficacy to achievement.

Second, no direct measure of general intelligence, working memory, or another domain-general cognitive ability was available. Mathematics performance was treated as a domain-specific cognitive criterion, not as a proxy for intelligence. The analyses therefore cannot determine whether the experience–belief relations differ across levels of cognitive ability or whether particular cognitive resources strengthen the translation of experience into capability judgment. Future research should combine direct ability measures, mathematics-specific skills, task-level self-efficacy, and learning-process data in the same design.

Third, the learning experiences are student reports. This is theoretically relevant because self-efficacy not only depends on interpreted experience but also raises concerns about shared-method variance, response style, and classroom reference frames. The concern is especially important for the direct task-exposure contrasts. The exposure items ask how often students encounter particular forms of mathematical work, whereas the self-efficacy items ask whether students believe they can perform related work. The judgments are therefore different, but the matched measures intentionally share mathematical content. That shared referent is necessary for testing content correspondence and may also strengthen covariance independently of the proposed interpretation. Replication across education systems does not eliminate this possibility because the same reporting method is used in every system. Observations, teacher reports, lesson materials, performance traces, and digital task histories could help separate enacted opportunities from students’ reports of them. The item-level MGCFA supports construct distinguishability but does not replace the PISA’s international IRT scaling or establish invariance of the published WLE scores (OECD, 2024a, 2024b).

Fourth, informational proximity is an organizing proposition inferred from theoretically distinct classes of experience; it was not directly measured or experimentally manipulated. This study therefore cannot rule out semantic or content-overlap explanations for the especially strong direct task-matching pattern. The evidence is consistent with the proposed ordering and is not a direct test of proximity as a psychological mechanism or a universal monotonic gradient. The ordered-categorical WLSMV sensitivity recovered the intended factor structures, although absolute fit was mixed for task exposure and digital inquiry under the deliberately conservative no-residual specification. The no-residual MLR sensitivity likewise showed that recurrent local dependence materially improves fit and that exact loading invariance is more sensitive than construct differentiation. These qualifications reinforce the narrower interpretation of the item-level CFA as complementary evidence about construct organization rather than a reconstruction or validation of the official PISA WLE scales.

Fifth, RQ3 used overall PISA mathematics performance as a broad domain-specific criterion. This choice matches the study’s question about external mathematics performance, but it may mask finer criterion correspondence. The two questionnaire self-efficacy dimensions distinguish broad classes of mathematical task content, whereas the PISA process scores partition assessed performance by formulating, employing, interpreting/evaluating, and reasoning processes; these classifications do not map one to one. Process-specific plausible values or task-level outcomes could therefore yield a different pattern and should be tested in a prespecified extension rather than interpreted as a direct validation of the present two self-efficacy dimensions. Methodologically, the analyses used the final student weight, school clustering, robust estimation, and all plausible values but did not use the 80 replicate weights because they were absent from the analytic files used for the structural models. This primarily limits the precision of system-specific standard errors, *p* values, and significance classifications. It is less consequential for the weighted point estimates themselves, but the exact counts of significant systems should be interpreted cautiously. The primary structural model was saturated, so inference rests on theory-specified contrasts, coefficient magnitude and direction, cross-system replication, random-effects synthesis, and prediction intervals rather than global model fit.

## 6. Conclusions

Across 49 education systems, mathematics self-efficacy showed a differentiated within-domain structure, and learning experiences corresponded to that structure in theoretically meaningful ways. Direct task exposure produced the clearest content matching: formal/applied and reasoning/21st-century experiences were more strongly associated with capability belief that referred to similar demands, and the combined task-matching pattern was positive in every system. A scale-free sensitivity based on standardized pathways showed the same combined direction in all 49 systems. Cognitive activation showed differentiated but nonliteral correspondence, while digital inquiry was broadly positive and more context-dependent. These findings are consistent with the proposed informational-proximity ordering, in which experiences that more closely instantiate the capability being judged provide more directly corresponding information for self-efficacy.

The findings also clarify the place of self-efficacy in research on mathematical cognition. Self-efficacy is neither intelligence nor performance. It is a task-referenced appraisal that accompanies the use and development of knowledge and cognitive resources. Closely related beliefs can share a common mathematics-capability component while differing in the experiences associated with them and in their correspondence with a cognitive performance criterion. The main contribution is therefore not simply that learning experiences relate to confidence. It is that the organization, specificity, and cross-system generalizability of those relations depend on what students have been asked to do and on what capability they are judging.

## Figures and Tables

**Table 1:** The focal constructs and roles in the analysis.

Code	Construct	Role	Operational Meaning
ICTENQ	ICT use in inquiry-based learning	Learning experience	Reported use of digital tools to formulate questions, investigate, and develop understanding
COGACRCO	Cognitive activation: foster reasoning	Learning experience	Instructional experiences that prompt reasoning, explanation, and cognitively demanding engagement
COGACMCO	Cognitive activation: encourage mathematical thinking	Learning experience	Instructional experiences that encourage students to think about mathematical ideas and processes
EXPOFA	Exposure to formal and applied mathematics tasks	Learning experience	Frequency of encountering formal or applied mathematics tasks
EXPO21ST	Exposure to mathematical reasoning and 21st-century tasks	Learning experience	Frequency of encountering reasoning, modeling, data, or other 21st-century mathematics tasks
MATHEFF	Self-efficacy for formal and applied mathematics	Task-specific self-belief	Perceived capability for formal and applied mathematics tasks
MATHEF21	Self-efficacy for reasoning and 21st-century mathematics	Task-specific self-belief	Perceived capability for mathematical reasoning and 21st-century mathematics tasks
PVMATH	Mathematics plausible values	Domain-specific cognitive outcome	Ten plausible values representing performance in PISA mathematics assessment; not direct measure of general intelligence
ESCS	Economic, social and cultural status	Covariate	Composite index of family socioeconomic and cultural resources
Gender	Student gender	Covariate	Binary student-level covariate retained from analytic dataset

Note: The construct labels and descriptions follow the PISA 2022 assessment and questionnaire frameworks. The structural analyses used the published PISA indices. Item-level analyses evaluated factor structure and loading comparability but were not used to reconstruct, replace, or establish invariance of the official PISA WLE scores. PVMATH denotes the ten mathematics plausible values rather than a directly observed scale score.

**Table 2:** Random-effects synthesis of formal within-system contrasts.

Contrast	Pooled Difference	95% CI	95% Prediction Interval	I^2^ (%)	Significant +/− Systems
Digital inquiry: MATHEF21—MATHEFF	0.030	[0.019, 0.040]	[−0.029, 0.088]	65.5	18/1
Cognitive activation—foster reasoning: MATHEF21—MATHEFF	−0.062	[−0.073, −0.052]	[−0.119, −0.005]	54.6	0/34
Cognitive activation—encourage mathematical thinking: MATHEF21—MATHEFF	0.072	[0.058, 0.085]	[−0.006, 0.149]	66.7	32/0
Formal/applied task exposure: MATHEFF—MATHEF21	0.078	[0.063, 0.092]	[−0.008, 0.164]	73.8	32/0
Reasoning/21st-century task exposure: MATHEF21—MATHEFF	0.120	[0.109, 0.131]	[0.063, 0.176]	55.2	48/0
Overall task-matching index	0.099	[0.089, 0.109]	[0.046, 0.152]	55.8	44/0

Note. k = 49 education systems. MATHEFF—self-efficacy for formal/applied mathematics tasks; MATHEF21—self-efficacy for reasoning/21st-century mathematics tasks. Pooled differences are unstandardized within-system path contrasts calculated with the covariance between paired coefficients. The digital-inquiry and cognitive-activation contrasts are coded as MATHEF21 minus MATHEFF. The formal/applied task-exposure contrast is coded as MATHEFF minus MATHEF21, whereas the reasoning/21st-century task-exposure contrast is coded as MATHEF21 minus MATHEFF. The overall task-matching summary is the mean of the two task-exposure contrasts after both are oriented as matched minus unmatched. I^2^ indicates the percentage of variability in system-specific estimates attributable to between-system heterogeneity rather than sampling error. “Significant +/− systems” reports the numbers of education systems with statistically significant positive and negative contrasts, respectively, according to the coding shown in the first column. Confidence intervals are based on Hartung–Knapp random-effects inference.

**Table 3:** Pooled standardized associations of learning experiences and socioeconomic status with the two self-efficacy dimensions.

Predictor	MATHEFF Beta [95% CI]	MATHEF21 Beta [95% CI]	Significant Positive Systems MATHEFF/MATHEF21	I^2^ (%) MATHEFF/MATHEF21
Digital inquiry	0.074 [0.060, 0.087]	0.117 [0.107, 0.128]	35/47	73.1/52.6
Cognitive activation—foster reasoning	0.111 [0.099, 0.123]	0.066 [0.057, 0.076]	46/31	57.9/34.8
Cognitive activation—encourage mathematical thinking	0.072 [0.055, 0.089]	0.155 [0.142, 0.167]	32/48	77.5/61.7
Formal/applied task exposure	0.074 [0.059, 0.089]	0.014 [0.004, 0.023]	30/5	72.6/39.2
Reasoning/21st-century task exposure	0.018 [0.005, 0.030]	0.141 [0.128, 0.154]	8/46	59.1/63.1
ESCS	0.204 [0.188, 0.221]	0.125 [0.111, 0.139]	48/47	87.2/82.7

Note. k = 49 education systems. MATHEFF—self-efficacy for formal/applied mathematics tasks; MATHEF21—self-efficacy for reasoning/21st-century mathematics tasks; ESCS—index of economic, social and cultural status. β values are pooled standardized (STDYX) coefficients from random-effects models and describe the magnitude of adjusted associations. All five learning experiences, the ESCS, and gender were entered simultaneously in the system-specific models. “Significant positive systems MATHEFF/MATHEF21” reports the numbers of education systems in which the corresponding association was positive and statistically significant for MATHEFF and MATHEF21, respectively. I^2^ values are reported in the same MATHEFF/MATHEF21 order and indicate the percentage of variability attributable to between-system heterogeneity rather than sampling error. Confidence intervals are based on Hartung–Knapp random-effects inference. Formal paired-path contrasts between MATHEFF and MATHEF21 are reported in Table 2.

**Table 4:** Cross-system synthesis of self-efficacy–performance correspondence and explained variance.

Model Coefficient	Pooled Beta	95% CI	95% Prediction Interval	Significant +/− Systems	Mean PVMATH R^2^ (ΔR^2^)
MATHEFF entered alone	0.377	[0.351, 0.403]	[0.201, 0.553]	49/0	0.301 (+0.130)
MATHEF21 entered alone	0.218	[0.197, 0.240]	[0.072, 0.365]	48/0	0.215 (+0.044)
MATHEFF in joint model	0.394	[0.369, 0.420]	[0.223, 0.565]	49/0	0.302 (+0.131; joint)
MATHEF21 in joint model	−0.032	[−0.043, −0.021]	[−0.087, 0.023]	1/13	0.302 (+0.131; joint)

Note. k = 49 education systems. MATHEFF—self-efficacy for formal/applied mathematics tasks; MATHEF21—self-efficacy for reasoning/21st-century mathematics tasks; PVMATH—mathematics performance represented by the ten plausible values. β values are pooled standardized (STDYX) coefficients and describe effect magnitude. “Significant +/− systems” reports the numbers of education systems with statistically significant positive and negative coefficients, respectively. The baseline model included the five learning experiences, the ESCS, and gender and had a mean PVMATH R^2^ of 0.171 across the 49 education systems. The separate models added MATHEFF or MATHEF21 individually, whereas the joint model included both self-efficacy dimensions simultaneously. Joint-model coefficients are therefore conditional on the other self-efficacy dimension. Mean PVMATH R^2^ is the mean of system-specific explained-variance estimates; ΔR^2^ is the change from the baseline mean R^2^. Confidence intervals are based on Hartung–Knapp random-effects inference.

## Data Availability

This study used public-use data from the OECD Programme for International Student Assessment (PISA) 2022. The raw student data files, questionnaires, codebooks, and technical documentation are publicly available from the OECD PISA 2022 Database at: https://www.oecd.org/en/data/datasets/pisa-2022-database.html (accessed on 3 October 2026).

## Supplementary Materials

The following supporting information can be downloaded at: https://www.mdpi.com/article/10.3390/jintelligence14100263/s1: Table S1, Education systems, analytic sample sizes, and school clusters; Table S2, System-specific primary-model contrasts and omnibus Wald tests; Table S3, Complete random-effects synthesis of standardized primary-model pathways; Table S4, System-specific self-efficacy performance coefficients and explained variance; Table S5, System-specific residual correlations and primary-model explained variance; Table S6, Item-level MGCFA model fit, construct distinguishability, and measurement-invariance results; Table S7, Retained residual covariances in item-level measurement models; Table S8, System-specific scale-free task-matching sensitivity based on STDYX coefficients; Table S9, Cross-system synthesis of residual correlations among five learning-experience predictors; Table S10, Ordered-categorical WLSMV sensitivity of intended measurement structures; Table S11, MLR sensitivity without retained residual covariances.
