# Supplementary material for: When Mathematical Learning Experiences Match Self-Efficacy Beliefs: Content Specificity and Cognitive Performance Across 49 Education Systems
Source: J Intell. 2026 Oct 16;14(10):263. doi: 10.3390/jintelligence14100263 (PMC13705892; doi:10.3390/jintelligence14100263)
Supplement: Supplementary file 1 [file jintelligence-14-00263-s001.zip › jintelligence-4554678-supplementary.pdf]

Supplementary Material

When Mathematical Learning Experiences Match Self-Efficacy Beliefs: Content Specificity and Cognitive Performance across 49 Education Systems

Table S1. Education systems, analytic sample sizes, and school clusters.

Code	Education system	Students	Schools
ALB	Albania	2782	258
ARG	Argentina	5870	450
AUS	Australia	6484	739
AUT	Austria	2996	296
BEL	Belgium	4012	283
BRA	Brazil	5167	581
BRN	Brunei Darussalam	2769	53
BGR	Bulgaria	2926	201
CHL	Chile	3086	219
TPE	Chinese Taipei	2902	182
HRV	Croatia	3038	179
CZE	Czech Republic	4141	425
DNK	Denmark	2976	342
DOM	Dominican Republic	3373	249
EST	Estonia	3139	191
FIN	Finland	4965	239
GEO	Georgia	3108	263
DEU	Germany	2661	252
GRC	Greece	3151	228
HKG	Hong Kong China	2808	163
HUN	Hungary	3009	251
ISL	Iceland	1642	127
IRL	Ireland	2766	170
ISR	Israel	3009	193
ITA	Italy	5221	338
JPN	Japan	2850	182

Code	Education system	Students	Schools
JOR	Jordan	3831	259
KOR	Korea	3198	186
LVA	Latvia	2598	223
LTU	Lithuania	3524	282
MAC	Macao China	2190	46
MYS	Malaysia	3504	198
MLT	Malta	1487	45
MAR	Morocco	3420	177
PAN	Panama	2114	201
POL	Poland	2933	237
ROU	Romania	3645	255
SAU	Saudi Arabia	3396	193
SGP	Singapore	3283	164
SVK	Slovak Republic	2878	282
SVN	Slovenia	3300	331
ESP	Spain	14819	959
SWE	Sweden	2938	256
CHE	Switzerland	3293	254
TUR	Turkiye	3621	196
GBR	United Kingdom	5552	442
USA	United States	2169	148
URY	Uruguay	3257	220
UKR	Ukrainian regions	1840	160

Note. The analytic sample comprised 173,641 students across 49 education systems. Counts correspond to the system-specific primary-model analytic samples.

Table S2. System-specific primary-model contrasts and omnibus Wald tests.

Code	Omnibus Wald χ^2 (p)	D_{ICT} est. (p)	D_{RCO} est. (p)	D_{MCO} est. (p)	D_{EFA} est. (p)	D_{E21} est. (p)	TASKMAT est. (p)
ALB	22.716 (<.001)	0.015 (.596)	0.001 (.969)	-0.056 (.077)	0.148 (<.001)	0.128 (<.001)	0.138 (<.001)
ARG	43.734 (<.001)	0.004 (.876)	-0.067 (.011)	0.097 (.001)	0.037 (.222)	0.119 (<.001)	0.078 (.002)

Code	Omnibus Wald χ^2 (p)	D_{ICT} est. (p)	D_{RCO} est. (p)	D_{MCO} est. (p)	D_{EFA} est. (p)	D_{E21} est. (p)	TASKMAT est. (p)
AUS	56.745 (<.001)	0.028 (.057)	-0.043 (.012)	0.045 (.007)	0.124 (<.001)	0.076 (<.001)	0.100 (<.001)
AUT	103.324 (<.001)	0.102 (<.001)	-0.064 (.005)	0.140 (<.001)	0.160 (<.001)	0.137 (<.001)	0.148 (<.001)
BEL	92.387 (<.001)	0.071 (.003)	-0.093 (<.001)	0.086 (<.001)	0.066 (.016)	0.144 (<.001)	0.105 (<.001)
BRA	26.352 (<.001)	0.035 (.046)	-0.052 (.030)	0.042 (.052)	0.067 (.004)	0.076 (.001)	0.072 (<.001)
BRN	39.930 (<.001)	0.016 (.308)	-0.045 (.053)	0.092 (<.001)	0.113 (<.001)	0.122 (<.001)	0.117 (<.001)
BGR	40.915 (<.001)	0.022 (.492)	-0.123 (<.001)	0.122 (<.001)	0.044 (.195)	0.079 (.004)	0.061 (.023)
CHL	25.261 (<.001)	-0.005 (.852)	0.003 (.913)	-0.018 (.590)	0.101 (<.001)	0.139 (<.001)	0.120 (<.001)
TPE	30.850 (<.001)	0.004 (.827)	0.017 (.450)	0.067 (.005)	0.069 (.003)	0.065 (.012)	0.067 (.003)
HRV	35.938 (<.001)	0.028 (.160)	-0.031 (.136)	0.057 (.014)	0.050 (.035)	0.098 (<.001)	0.074 (.001)
CZE	112.431 (<.001)	0.052 (.020)	-0.074 (<.001)	0.070 (.006)	0.136 (<.001)	0.174 (<.001)	0.155 (<.001)
DNK	22.055 (<.001)	0.034 (.170)	-0.067 (.005)	0.049 (.083)	0.091 (.001)	0.052 (.042)	0.071 (.002)
DOM	8.531 (.129)	-0.041 (.100)	-0.022 (.537)	0.054 (.214)	0.009 (.814)	0.072 (.065)	0.040 (.247)
EST	85.619 (<.001)	0.068 (.001)	-0.092 (.002)	0.090 (.004)	0.092 (<.001)	0.176 (<.001)	0.134 (<.001)
FIN	108.003 (<.001)	0.003 (.886)	-0.021 (.354)	0.062 (.007)	0.193 (<.001)	0.158 (<.001)	0.175 (<.001)
GEO	19.398 (.002)	0.015 (.569)	-0.078 (.006)	0.014 (.636)	0.042 (.152)	0.094 (.002)	0.068 (.008)
DEU	84.526 (<.001)	0.046 (.118)	-0.149 (<.001)	0.162 (<.001)	0.126 (<.001)	0.147 (<.001)	0.137 (<.001)
GRC	122.027 (<.001)	0.060 (.002)	-0.166 (<.001)	0.153 (<.001)	0.039 (.137)	0.124 (<.001)	0.081 (<.001)
HKG	58.795 (<.001)	0.022 (.239)	-0.073 (.003)	0.100 (<.001)	0.059 (.052)	0.162 (<.001)	0.111 (<.001)
HUN	111.452 (<.001)	0.076 (.002)	-0.152 (<.001)	0.105 (<.001)	0.065 (.027)	0.179 (<.001)	0.122 (<.001)
ISL	43.468 (<.001)	0.078 (.027)	-0.046 (.304)	0.058 (.142)	0.087 (.053)	0.186 (<.001)	0.137 (<.001)
IRL	69.229 (<.001)	0.037 (.049)	-0.057 (.010)	0.065 (.001)	0.167 (<.001)	0.162 (<.001)	0.165 (<.001)
ISR	111.894 (<.001)	0.104 (.003)	-0.107 (<.001)	0.098 (.001)	0.020 (.358)	0.144 (<.001)	0.082 (<.001)
ITA	116.423 (<.001)	0.030 (.197)	-0.055 (.018)	0.093 (<.001)	0.002 (.907)	0.160 (<.001)	0.081 (<.001)
JPN	66.791 (<.001)	-0.014 (.512)	-0.055 (.009)	0.122 (<.001)	0.111 (<.001)	0.123 (<.001)	0.117 (<.001)
JOR	23.855 (<.001)	0.026 (.154)	-0.017 (.607)	-0.011 (.714)	0.061 (.025)	0.132 (<.001)	0.096 (<.001)
KOR	60.826 (<.001)	-0.066 (<.001)	-0.051 (.024)	0.100 (<.001)	0.066 (<.001)	0.120 (<.001)	0.093 (<.001)
LVA	46.831 (<.001)	0.083 (.001)	-0.039 (.194)	-0.006 (.839)	-0.034 (.184)	0.122 (<.001)	0.044 (.052)
LTU	60.438 (<.001)	0.014 (.470)	-0.102 (<.001)	0.101 (<.001)	0.007 (.794)	0.117 (<.001)	0.062 (.005)
MAC	35.137 (<.001)	-0.037 (.127)	-0.069 (.008)	0.060 (.073)	0.050 (.092)	0.125 (<.001)	0.087 (<.001)

Code	Omnibus Wald χ^2 (p)	D_{ICT} est. (p)	D_{RCO} est. (p)	D_{MCO} est. (p)	D_{EFA} est. (p)	D_{E21} est. (p)	TASKMAT est. (p)
MYS	24.945 (<.001)	-0.002 (.850)	-0.045 (.024)	0.035 (.083)	0.069 (.001)	0.065 (<.001)	0.067 (<.001)
MLT	50.998 (<.001)	0.071 (.008)	-0.080 (<.001)	0.088 (.002)	0.159 (<.001)	0.109 (.006)	0.134 (<.001)
MAR	13.736 (.017)	-0.006 (.782)	-0.046 (.096)	0.051 (.071)	0.066 (.070)	0.101 (.001)	0.084 (.006)
PAN	11.399 (.044)	0.001 (.972)	-0.054 (.122)	0.030 (.428)	0.039 (.397)	0.109 (.008)	0.074 (.050)
POL	84.969 (<.001)	0.059 (.019)	-0.095 (.001)	0.134 (<.001)	0.023 (.440)	0.126 (<.001)	0.075 (.003)
ROU	38.754 (<.001)	0.033 (.127)	-0.076 (.001)	0.028 (.254)	0.060 (.014)	0.127 (<.001)	0.094 (<.001)
SAU	34.191 (<.001)	-0.003 (.862)	-0.049 (.021)	-0.028 (.189)	0.081 (.002)	0.102 (<.001)	0.092 (<.001)
SGP	50.985 (<.001)	0.031 (.135)	-0.038 (.081)	0.112 (<.001)	0.089 (<.001)	0.050 (.029)	0.070 (.001)
SVK	43.805 (<.001)	0.031 (.181)	-0.045 (.049)	0.077 (.001)	0.124 (<.001)	0.105 (<.001)	0.115 (<.001)
SVN	60.884 (<.001)	0.095 (.001)	-0.098 (.002)	0.114 (<.001)	0.020 (.521)	0.087 (.016)	0.053 (.080)
ESP	137.701 (<.001)	0.028 (.094)	-0.036 (.040)	0.040 (.010)	0.086 (<.001)	0.178 (<.001)	0.132 (<.001)
SWE	69.684 (<.001)	0.052 (.014)	-0.109 (<.001)	0.084 (.002)	0.075 (.007)	0.138 (<.001)	0.107 (<.001)
CHE	78.559 (<.001)	0.087 (.002)	-0.067 (.017)	0.127 (<.001)	0.107 (<.001)	0.155 (<.001)	0.131 (<.001)
TUR	38.880 (<.001)	0.070 (<.001)	-0.032 (.148)	0.031 (.208)	0.082 (.001)	0.082 (<.001)	0.082 (<.001)
GBR	83.524 (<.001)	0.014 (.519)	-0.076 (.003)	0.132 (<.001)	0.167 (<.001)	0.171 (<.001)	0.169 (<.001)
USA	33.425 (<.001)	0.043 (.045)	-0.012 (.565)	0.074 (.002)	0.102 (<.001)	0.058 (.026)	0.080 (<.001)
URY	61.221 (<.001)	0.043 (.069)	-0.074 (.012)	0.103 (.001)	0.109 (.001)	0.187 (<.001)	0.148 (<.001)
UKR	17.285 (.004)	-0.028 (.462)	-0.087 (.008)	0.061 (.114)	-0.022 (.487)	0.063 (.039)	0.020 (.436)

Note. D_ICT, D_RCO, and D_MCO are coded MATHEF21 minus MATHEFF. D_EFA is coded MATHEFF minus MATHEF21, and D_E21 is coded MATHEF21 minus MATHEFF; thus positive D_EFA and D_E21 values indicate content matching. TASKMAT is the mean of D_EFA and D_E21. Formal contrasts are unstandardized within-system coefficient differences. p values are two-sided.

Table S3. Complete random-effects synthesis of standardized primary-model pathways.

Outcome	Predictor	Pooled β	95% CI	95% prediction interval	I^2 (%)	Significant +/- systems
MATHEFF	Digital inquiry	0.074	[0.060, 0.087]	[-0.010, 0.157]	73.1	35/0
MATHEFF	Cognitive activation: foster reasoning	0.111	[0.099, 0.123]	[0.047, 0.174]	57.9	46/0
MATHEFF	Cognitive activation: encourage mathematical thinking	0.072	[0.055, 0.089]	[-0.034, 0.178]	77.5	32/1
MATHEFF	Formal/applied task exposure	0.074	[0.059, 0.089]	[-0.018, 0.166]	72.6	30/0
MATHEFF	Reasoning/21st-century task exposure	0.018	[0.005, 0.030]	[-0.048, 0.083]	59.1	8/2

Outcome	Predictor	Pooled β	95% CI	95% prediction interval	I² (%)	Significant +/- systems
MATHEFF	ESCS	0.204	[0.188, 0.221]	[0.093, 0.316]	87.2	48/0
MATHEFF	Gender	0.091	[0.074, 0.109]	[-0.025, 0.207]	89.5	37/1
MATHEF21	Digital inquiry	0.117	[0.107, 0.128]	[0.065, 0.170]	52.6	47/0
MATHEF21	Cognitive activation: foster reasoning	0.066	[0.057, 0.076]	[0.026, 0.107]	34.8	31/0
MATHEF21	Cognitive activation: encourage mathematical thinking	0.155	[0.142, 0.167]	[0.084, 0.225]	61.7	48/0
MATHEF21	Formal/applied task exposure	0.014	[0.004, 0.023]	[-0.030, 0.057]	39.2	5/2
MATHEF21	Reasoning/21st-century task exposure	0.141	[0.128, 0.154]	[0.066, 0.216]	63.1	46/0
MATHEF21	ESCS	0.125	[0.111, 0.139]	[0.038, 0.212]	82.7	47/0
MATHEF21	Gender	0.132	[0.113, 0.152]	[0.001, 0.264]	91.5	43/1
PVMATH	Digital inquiry	-0.033	[-0.048, -0.018]	[-0.127, 0.061]	78.7	3/20
PVMATH	Cognitive activation: foster reasoning	0.106	[0.092, 0.121]	[0.019, 0.194]	73.6	44/0
PVMATH	Cognitive activation: encourage mathematical thinking	-0.074	[-0.090, -0.058]	[-0.172, 0.025]	77.7	1/33
PVMATH	Formal/applied task exposure	0.046	[0.028, 0.064]	[-0.071, 0.163]	84.2	26/5
PVMATH	Reasoning/21st-century task exposure	-0.039	[-0.056, -0.022]	[-0.150, 0.072]	83.7	5/22
PVMATH	ESCS	0.359	[0.340, 0.379]	[0.227, 0.492]	88.5	49/0
PVMATH	Gender	0.046	[0.032, 0.061]	[-0.043, 0.136]	79.5	27/3

Note. Coefficients are pooled STDYX-standardized associations from system-specific primary models. All predictors were entered simultaneously within each outcome equation. The PVMATH pathways are conditional associations among correlated learning experiences and covariates; sign changes under mutual adjustment should not be interpreted as harmful effects. Random-effects synthesis used Paule-Mandel between-system variance estimation and Hartung-Knapp inference.

Table S4. System-specific self-efficacy-performance coefficients and explained variance.

Code	β MATHEFF only	β MATHEF21 only	β MATHEFF joint	β MATHEF21 joint	R^2 baseline	R^2 + MATHEFF	R^2 + MATHEF21	R^2 joint
ALB	0.299	0.210	0.276	0.034	0.099	0.176	0.135	0.176
ARG	0.296	0.144	0.327	-0.058	0.184	0.264	0.202	0.265
AUS	0.508	0.321	0.537	-0.042	0.165	0.379	0.248	0.380
AUT	0.395	0.239	0.403	-0.015	0.232	0.369	0.280	0.369
BEL	0.329	0.157	0.355	-0.048	0.239	0.338	0.260	0.339
BRA	0.246	0.091	0.315	-0.116	0.147	0.202	0.155	0.209
BRN	0.439	0.188	0.508	-0.116	0.179	0.350	0.210	0.358

Code	β MATHEFF only	β MATHEF21 only	β MATHEFF joint	β MATHEF21 joint	R^2 baseline	R^2 + MATHEFF	R^2 + MATHEF21	R^2 joint
BGR	0.267	0.164	0.275	-0.018	0.215	0.278	0.238	0.276
CHL	0.239	0.115	0.299	-0.089	0.145	0.197	0.157	0.201
TPE	0.464	0.301	0.500	-0.056	0.189	0.367	0.261	0.367
HRV	0.417	0.263	0.432	-0.026	0.164	0.319	0.222	0.318
CZE	0.426	0.206	0.460	-0.061	0.237	0.388	0.271	0.389
DNK	0.464	0.344	0.468	-0.006	0.139	0.316	0.232	0.316
DOM	0.090	0.042	0.099	-0.017	0.142	0.149	0.143	0.149
EST	0.448	0.269	0.447	0.003	0.158	0.334	0.218	0.334
FIN	0.466	0.338	0.456	0.015	0.154	0.339	0.247	0.340
GEO	0.362	0.243	0.340	0.034	0.111	0.228	0.164	0.228
DEU	0.415	0.168	0.461	-0.087	0.219	0.364	0.242	0.368
GRC	0.393	0.235	0.394	-0.003	0.162	0.298	0.210	0.298
HKG	0.415	0.285	0.447	-0.046	0.069	0.228	0.138	0.229
HUN	0.421	0.155	0.460	-0.080	0.281	0.433	0.302	0.436
ISL	0.441	0.273	0.471	-0.051	0.097	0.271	0.158	0.273
IRL	0.478	0.270	0.491	-0.025	0.154	0.349	0.214	0.349
ISR	0.375	0.208	0.409	-0.059	0.220	0.345	0.254	0.346
ITA	0.395	0.182	0.415	-0.041	0.171	0.310	0.198	0.311
JPN	0.453	0.274	0.467	-0.024	0.141	0.320	0.205	0.320
JOR	0.210	0.090	0.224	-0.031	0.090	0.129	0.097	0.129
KOR	0.481	0.386	0.428	0.077	0.164	0.352	0.281	0.355
LVA	0.423	0.276	0.409	0.025	0.164	0.322	0.228	0.323
LTU	0.395	0.191	0.441	-0.079	0.177	0.316	0.208	0.319
MAC	0.469	0.292	0.481	-0.019	0.079	0.275	0.149	0.275
MYS	0.330	0.203	0.350	-0.030	0.210	0.301	0.245	0.302
MLT	0.464	0.313	0.463	-0.001	0.119	0.304	0.200	0.304
MAR	0.272	0.138	0.272	-0.004	0.124	0.189	0.141	0.188
PAN	0.248	0.081	0.321	-0.123	0.214	0.269	0.219	0.279
POL	0.389	0.189	0.420	-0.058	0.185	0.323	0.215	0.324

Code	β MATHEFF only	β MATHEF21 only	β MATHEFF joint	β MATHEF21 joint	R^2 baseline	R^2 + MATHEFF	R^2 + MATHEF21	R^2 joint
ROU	0.260	0.140	0.277	-0.027	0.306	0.366	0.324	0.366
SAU	0.238	0.126	0.262	-0.036	0.079	0.127	0.092	0.128
SGP	0.449	0.254	0.468	-0.032	0.183	0.348	0.234	0.349
SVK	0.325	0.191	0.347	-0.036	0.256	0.347	0.287	0.347
SVN	0.342	0.180	0.359	-0.031	0.193	0.296	0.220	0.297
ESP	0.394	0.203	0.411	-0.033	0.155	0.294	0.190	0.294
SWE	0.464	0.346	0.433	0.044	0.176	0.365	0.278	0.365
CHE	0.425	0.219	0.458	-0.058	0.218	0.375	0.257	0.377
TUR	0.356	0.219	0.355	0.001	0.174	0.288	0.215	0.288
GBR	0.485	0.274	0.509	-0.040	0.142	0.341	0.202	0.342
USA	0.419	0.277	0.429	-0.014	0.163	0.312	0.225	0.313
URY	0.289	0.169	0.289	-0.003	0.211	0.288	0.236	0.287
UKR	0.349	0.220	0.348	-0.002	0.193	0.296	0.234	0.295

Note. All models retain the five learning experiences, socioeconomic status, and gender. "Baseline" denotes the primary performance equation without either self-efficacy dimension. The joint-model coefficients are conditional on the other self-efficacy dimension.

Table S5. System-specific residual correlations and primary-model explained variance.

Code	r MATHEFF-MATHEF21	r MATHEFF-PVMATH	r MATHEF21-PVMATH	R^2 MATHEFF	R^2 MATHEF21	R^2 PVMATH
ALB	0.646	0.290	0.207	0.142	0.169	0.099
ARG	0.589	0.311	0.145	0.093	0.164	0.184
AUS	0.669	0.507	0.316	0.170	0.188	0.165
AUT	0.609	0.422	0.247	0.121	0.155	0.232
BEL	0.560	0.361	0.167	0.086	0.133	0.239
BRA	0.632	0.252	0.089	0.091	0.133	0.147
BRN	0.597	0.456	0.195	0.114	0.115	0.179
BGR	0.645	0.278	0.169	0.126	0.146	0.215
CHL	0.670	0.246	0.116	0.105	0.139	0.145
TPE	0.702	0.467	0.300	0.166	0.200	0.189
HRV	0.653	0.430	0.265	0.108	0.148	0.164
CZE	0.571	0.444	0.211	0.170	0.202	0.237

Code	r MATHEFF-MATHEF21	r MATHEFF-PVMATH	r MATHEF21-PVMATH	R^2 MATHEFF	R^2 MATHEF21	R^2 PVMATH
DNK	0.724	0.453	0.326	0.177	0.210	0.139
DOM	0.556	0.091	0.039	0.116	0.138	0.142
EST	0.575	0.458	0.265	0.124	0.173	0.158
FIN	0.688	0.468	0.330	0.146	0.185	0.154
GEO	0.619	0.361	0.244	0.112	0.107	0.111
DEU	0.544	0.431	0.172	0.157	0.184	0.219
GRC	0.588	0.403	0.235	0.117	0.133	0.162
HKG	0.713	0.413	0.273	0.078	0.147	0.069
HUN	0.509	0.457	0.168	0.147	0.153	0.281
ISL	0.648	0.439	0.257	0.096	0.180	0.097
IRL	0.589	0.479	0.266	0.146	0.178	0.154
ISR	0.616	0.399	0.209	0.115	0.217	0.220
ITA	0.509	0.411	0.179	0.104	0.183	0.171
JPN	0.627	0.456	0.271	0.128	0.152	0.141
JOR	0.534	0.204	0.087	0.123	0.155	0.090
KOR	0.706	0.474	0.372	0.189	0.219	0.164
LVA	0.600	0.436	0.277	0.117	0.160	0.164
LTU	0.594	0.411	0.192	0.115	0.172	0.177
MAC	0.620	0.461	0.275	0.110	0.176	0.079
MYS	0.661	0.339	0.207	0.165	0.163	0.210
MLT	0.661	0.458	0.302	0.137	0.179	0.119
MAR	0.505	0.271	0.133	0.120	0.138	0.124
PAN	0.592	0.267	0.074	0.097	0.126	0.214
POL	0.567	0.410	0.193	0.094	0.157	0.185
ROU	0.603	0.293	0.157	0.123	0.110	0.306
SAU	0.632	0.229	0.124	0.151	0.147	0.079
SGP	0.601	0.450	0.251	0.179	0.205	0.183
SVK	0.641	0.350	0.201	0.132	0.145	0.256
SVN	0.567	0.358	0.181	0.117	0.151	0.193

Code	r MATHEFF-MATHEF21	r MATHEFF-PVMATH	r MATHEF21-PVMATH	R^2 MATHEFF	R^2 MATHEF21	R^2 PVMATH
ESP	0.553	0.405	0.201	0.105	0.159	0.155
SWE	0.684	0.477	0.350	0.121	0.140	0.176
CHE	0.581	0.448	0.222	0.130	0.174	0.218
TUR	0.596	0.372	0.223	0.097	0.140	0.174
GBR	0.602	0.482	0.265	0.155	0.197	0.142
USA	0.654	0.422	0.269	0.153	0.193	0.163
URY	0.554	0.310	0.170	0.085	0.141	0.211
UKR	0.634	0.355	0.224	0.151	0.147	0.193

Note. Residual correlations are estimated after adjustment for the learning experiences, socioeconomic status, and gender in the primary model. R^2 values are system-specific.

Table S6. Item-level multigroup CFA model fit, construct distinguishability, and measurement-invariance results.

Measurement block	Model	N	χ^2 (df)	CFI	TLI	RMSEA	SRMR	ΔCFI	ΔRMSEA	ΔSRMR
Self-efficacy	One-factor comparison	308,282	103,214.292 (7,203)	.889	.871	.046	.071	—	—	—
	Configural (two-factor)	308,282	40,556.811 (7,154)	.961	.955	.027	.046	—	—	—
	Metric	308,282	46,166.311 (7,970)	.956	.954	.028	.056	-.005	.001	.010
	Scalar	308,282	85,589.727 (8,786)	.911	.916	.037	.073	-.045	.009	.017
Task exposure	One-factor comparison	310,823	114,339.765 (7,154)	.847	.820	.049	.081	—	—	—
	Configural (two-factor)	310,823	51,550.665 (7,105)	.936	.925	.031	.059	—	—	—
	Metric	310,823	61,667.907 (7,921)	.923	.919	.033	.076	-.013	.002	.017
	Scalar	310,823	110,984.497 (8,737)	.854	.860	.043	.090	-.069	.010	.014
Cognitive activation	One-factor comparison	316,018	172,705.704 (6,223)	.818	.781	.064	.103	—	—	—
	Configural (two-factor)	316,018	56,529.055 (6,174)	.945	.933	.036	.060	—	—	—
	Metric	316,018	64,310.625 (6,942)	.937	.932	.036	.067	-.008	.000	.007
	Scalar	316,018	105,483.378 (7,710)	.893	.896	.044	.078	-.044	.008	.011
Digital inquiry	Configural (one-factor)	308,080	28,256.757 (1,470)	.967	.951	.054	.030	—	—	—
	Metric	308,080	35,840.059 (1,902)	.959	.952	.053	.048	-.008	-.001	.018
	Scalar	308,080	71,441.174 (2,334)	.916	.921	.069	.081	-.043	.016	.033

Note. N = 308,282 for self-efficacy, 310,823 for task exposure, 316,018 for cognitive activation, and 308,080 for digital inquiry. For the paired blocks, one-factor alternatives are reported to evaluate construct distinguishability. Configural models use the final factor and residual-covariance structure with loadings and intercepts free across systems. Metric models constrain factor loadings across the 49 systems; scalar models additionally constrain item intercepts. Fit changes for metric models are calculated relative to configural models, and changes for scalar models are calculated relative to metric models. Conventional guides were considered jointly rather than as rigid cutoffs (Chen, 2007; Rutkowski & Svetina, 2014). The prespecified fit-change guides supported metric invariance for self-efficacy, cognitive activation, and digital inquiry. Task exposure did not meet the prespecified ΔCFI criterion for full metric invariance (ΔCFI = -.013), although ΔRMSEA = .002, ΔSRMR = .017, and absolute metric-model fit remained comparatively stable. We therefore interpret task exposure as showing a stable two-factor organization with some cross-system loading noninvariance rather than full metric invariance. Full scalar invariance was not supported for any block. No partial scalar models were pursued because latent mean

comparisons were outside the scope of the study. Latent correlations in the configural two-factor models ranged from .635 to .886 for self-efficacy (median = .758), .590 to .722 for task exposure (median = .658), and .456 to .727 for cognitive activation (median = .615).

Table S7. Retained residual covariances in the item-level measurement models.

Measurement block	Residual covariance	Substantive overlap
MATHEFF	ST290Q05WA WITH ST290Q07WA	Shared equation-solving task format
MATHEFF	ST290Q05WA WITH ST290Q09WA	Shared equation-solving task format
MATHEFF	ST290Q07WA WITH ST290Q09WA	Shared equation-solving task format
MATHEF21	ST291Q08JA WITH ST291Q09JA	Coding/programming and computer mathematics systems
MATHEF21	ST291Q02JA WITH ST291Q04JA	Real-life problem interpretation and identification
EXPOFA	ST275Q05WA WITH ST275Q07WA	Shared exposure to equation-solving tasks
EXPOFA	ST275Q05WA WITH ST275Q09WA	Shared exposure to equation-solving tasks
EXPOFA	ST275Q07WA WITH ST275Q09WA	Shared exposure to equation-solving tasks
EXPO21ST	ST276Q08JA WITH ST276Q09JA	Programming and computer mathematics systems
EXPO21ST	ST276Q02JA WITH ST276Q04JA	Real-life challenge and problem identification
EXPO21ST	ST276Q01JA WITH ST276Q06JA	Extracting and representing mathematical information
COGACRCO	ST285Q02JA WITH ST285Q03JA	Explanation of solutions and underlying assumptions
COGACRCO	ST285Q06JA WITH ST285Q07JA	Connections among ideas and alternative solution approaches
COGACRCO	ST285Q07JA WITH ST285Q08JA	Teacher prompts for sustained/non-routine engagement
COGACRCO	ST285Q08JA WITH ST285Q09JA	Adjacent teacher-practice items with shared response framing
COGACMCO	ST283Q03JA WITH ST283Q04JA	Thinking mathematically and using mathematical logic
COGACMCO	ST283Q04JA WITH ST283Q05JA	Mathematical logic and structural simplification
COGACMCO	ST283Q07JA WITH ST283Q09JA	Connections to larger mathematical ideas and contexts
COGACMCO	ST283Q01JA WITH ST283Q02JA	Everyday-life applicability of mathematics
ICTENQ	IC174Q04JA WITH IC174Q05JA	Collecting and analyzing data
ICTENQ	IC174Q07JA WITH IC174Q08JA	Planning/managing and tracking projects
ICTENQ	IC174Q01JA WITH IC174Q02JA	Digital production for school assignments
ICTENQ	IC174Q02JA WITH IC174Q03JA	Text/information work for school assignments
ICTENQ	IC174Q05JA WITH IC174Q06JA	Analysing and reporting investigation results

Note. Candidate local dependencies were screened with Mplus modification indices and STDYX expected parameter changes. The numerical values were used as pragmatic screening safeguards rather than universal cutoffs. An MI of at least 10 served as an initial flag. A residual covariance was retained only when the same item pair met that threshold in at least 40 of the 49 systems, the median absolute STDYX expected parameter change was at least .20 with a consistent direction, and the item content or response format provided a substantive explanation. The retained residual-covariance structure was held constant in the corresponding one- and two-factor comparisons and in the configural, metric, and scalar models; the residual covariance values themselves remained free across systems. Thus, factor-number comparisons did not favor one solution by giving it a different local-dependence specification.

Table S8. System-specific scale-free task-matching sensitivity based on STDYX coefficients.

Code	Education system	$D_{EFA,std}$	$D_{E21,std}$	$TASKMAT_{std}$
ALB	Albania	0.123	0.123	0.123
ARG	Argentina	0.032	0.130	0.081
AUS	Australia	0.086	0.092	0.089
AUT	Austria	0.135	0.138	0.136
BEL	Belgium	0.044	0.163	0.103
BGR	Bulgaria	0.047	0.083	0.065
BRA	Brazil	0.065	0.078	0.072
BRN	Brunei Darussalam	0.093	0.142	0.118
CHE	Switzerland	0.074	0.153	0.114
CHL	Chile	0.078	0.143	0.111
CZE	Czech Republic	0.095	0.157	0.126
DEU	Germany	0.080	0.135	0.108
DNK	Denmark	0.067	0.060	0.064
DOM	Dominican Republic	0.008	0.068	0.038
ESP	Spain	0.065	0.173	0.119
EST	Estonia	0.069	0.156	0.113
FIN	Finland	0.132	0.150	0.141
GBR	United Kingdom	0.119	0.180	0.149
GEO	Georgia	0.039	0.093	0.066
GRC	Greece	0.047	0.130	0.088
HKG	Hong Kong China	0.041	0.139	0.090
HRV	Croatia	0.038	0.104	0.071
HUN	Hungary	0.044	0.179	0.112
IRL	Ireland	0.130	0.162	0.146
ISL	Iceland	0.068	0.174	0.121
ISR	Israel	0.021	0.146	0.084
ITA	Italy	0.000	0.177	0.088
JOR	Jordan	0.049	0.127	0.088
JPN	Japan	0.087	0.141	0.114
KOR	Korea	0.061	0.117	0.089
LTU	Lithuania	0.005	0.126	0.066
LVA	Latvia	-0.033	0.118	0.043
MAC	Macao China	0.037	0.124	0.081
MAR	Morocco	0.053	0.091	0.072

Code	Education system	$D_{EFA,std}$	$D_{E21,std}$	$TASKMAT_{std}$
MLT	Malta	0.123	0.120	0.121
MYS	Malaysia	0.060	0.083	0.072
PAN	Panama	0.036	0.131	0.084
POL	Poland	0.026	0.124	0.075
ROU	Romania	0.048	0.118	0.083
SAU	Saudi Arabia	0.074	0.106	0.090
SGP	Singapore	0.062	0.074	0.068
SVK	Slovak Republic	0.103	0.099	0.101
SVN	Slovenia	0.010	0.086	0.048
SWE	Sweden	0.055	0.126	0.090
TPE	Chinese Taipei	0.041	0.083	0.062
TUR	Turkiye	0.057	0.087	0.072
UKR	Ukrainian regions	-0.024	0.068	0.022
URY	Uruguay	0.083	0.180	0.132
USA	United States	0.088	0.073	0.080

Note. D_EFA,std = β(EXPOFA→MATHEFF) − β(EXPOFA→MATHEF21); D_E21,std = β(EXPO21ST→MATHEF21) − β(EXPO21ST→MATHEFF); TASKMAT,std is their mean. Positive values indicate stronger matched-domain task pathways. These values are calculated from system-specific STDYX point estimates and are used only as a scale-free directional sensitivity check. Formal standard errors and p values are not reported because the covariance between the paired standardized coefficients is not available from the extracted STDYX output. The primary inferential contrasts therefore remain the unstandardized within-model differences in Table S2.

Table S9. Cross-system synthesis of residual correlations among the five learning-experience predictors.

Predictor 1	Predictor 2	Pooled r	95% CI	95% prediction interval	I² (%)	Interpretive role
ICTENQ	COGACRCO	0.132	[0.122, 0.142]	[0.085, 0.178]	46.1	Predictor-overlap diagnostic
ICTENQ	COGACMCO	0.181	[0.173, 0.189]	[0.157, 0.205]	18.7	Predictor-overlap diagnostic
ICTENQ	EXPOFA	0.093	[0.079, 0.108]	[0.007, 0.178]	68.3	Predictor-overlap diagnostic
ICTENQ	EXPO21ST	0.147	[0.132, 0.163]	[0.053, 0.239]	73.3	Predictor-overlap diagnostic
COGACRCO	COGACMCO	0.542	[0.530, 0.553]	[0.466, 0.609]	75.1	Predictor-overlap diagnostic
COGACRCO	EXPOFA	0.091	[0.075, 0.107]	[-0.006, 0.187]	74.4	Predictor-overlap diagnostic
COGACRCO	EXPO21ST	0.151	[0.132, 0.170]	[0.030, 0.268]	82.1	Predictor-overlap diagnostic
COGACMCO	EXPOFA	0.140	[0.124, 0.157]	[0.039, 0.239]	76.2	Predictor-overlap diagnostic
COGACMCO	EXPO21ST	0.275	[0.254, 0.296]	[0.131, 0.408]	87.4	Predictor-overlap diagnostic
EXPOFA	EXPO21ST	0.517	[0.504, 0.529]	[0.436, 0.589]	78.2	Predictor-overlap diagnostic

Note. Residual correlations were extracted from the primary system-specific models after adjustment for ESCS and gender, because the five learning-experience variables were modeled as endogenous outcomes of those covariates. System-specific correlations were transformed to Fisher z, synthesized with random-effects models, and back-transformed to r. The two largest pooled correlations were between the two cognitive-activation indices ($r = .542$) and between the two task-exposure indices ($r = .517$); the remaining pooled correlations ranged from .091 to .275. These results indicate meaningful shared variance without near-redundancy and are used to interpret mutually adjusted performance coefficients.

Table S10. Ordered-categorical WLSMV sensitivity of the intended measurement structures.

Measurement block	Intended model	CFI	TLI	RMSEA	SRMR
Self-efficacy	Two-factor configural	.971	.967	.061	.084
Task exposure	Two-factor configural	.934	.925	.068	.142
Cognitive activation	Two-factor configural	.982	.979	.051	.057
Digital inquiry	One-factor configural	.937	.919	.131	.042

Note. All four models treated the four- and five-category indicators as ordered categorical and used WLSMV. To make this a conservative estimator sensitivity, the locally correlated residuals retained in the primary MLR specification were omitted. The purpose was to assess whether the intended factor structures remained recoverable under ordinal estimation, not to repeat the full metric/scalar invariance sequence. Absolute fit was mixed for task exposure (elevated SRMR) and digital inquiry (elevated RMSEA), so these models are interpreted as sensitivity evidence rather than replacement measurement models.

Table S11. MLR sensitivity without retained residual covariances.

Measurement block	Model	CFI	TLI	RMSEA	SRMR	ΔCFI
Self-efficacy	One-factor comparison	.789	.763	.062	.093	—
Self-efficacy	Configural two-factor	.883	.868	.047	.068	—
Self-efficacy	Metric two-factor	.875	.872	.046	.074	-.008
Task exposure	One-factor comparison	.675	.634	.069	.115	—
Task exposure	Configural two-factor	.775	.745	.058	.098	—
Task exposure	Metric two-factor	.754	.749	.057	.106	-.021
Cognitive activation	One-factor comparison	.762	.731	.071	.111	—
Cognitive activation	Configural two-factor	.922	.911	.041	.061	—
Cognitive activation	Metric two-factor	.913	.912	.041	.068	-.009
Digital inquiry	Configural one-factor	.910	.884	.083	.047	—
Digital inquiry	Metric one-factor	.895	.892	.080	.061	-.015

Note. These models retain the original MLR, final student weight, and school-clustering specification but remove all residual covariances listed in Table S7. Removing local-dependence terms reduced absolute fit, especially for task exposure and digital inquiry. The two-factor configurations remained descriptively better than one-factor alternatives for self-efficacy, task exposure, and cognitive activation. The auxiliary no-residual one-factor models terminated normally but produced possible-multiple-solution warnings; their fit indices are therefore used descriptively only. Metric ΔCFI remained within .010 for self-efficacy and cognitive activation but exceeded .010 for task exposure and digital inquiry, indicating that exact loading invariance is more sensitive to the treatment of recurrent local dependence than is the basic dimensional distinction.
